# Recent progress in treatment of dyes wastewater using microbial-electro-Fenton technology

**DOI:** 10.1039/d2ra01831d

**Published:** 2022-06-09

**Authors:** Shumaila Rafaqat, Naeem Ali, Cesar Torres, Bruce Rittmann

**Affiliations:** Department of Microbiology, Quaid-i-Azam University Islamabad Pakistan; Department of Microbiology, Faculty of Biological Sciences, Quaid-i-Azam University Islamabad Pakistan naeemali@qau.edu.pk; Biodesign Swette Center for Environmental Biotechnology, Arizona State University USA

## Abstract

Globally, textile dyeing and manufacturing are one of the largest industrial units releasing huge amount of wastewater (WW) with refractory compounds such as dyes and pigments. Currently, wastewater treatment has been viewed as an industrial opportunity for rejuvenating fresh water resources and it is highly required in water stressed countries. This comprehensive review highlights an overall concept and in-depth knowledge on integrated, cost-effective cross-disciplinary solutions for domestic and industrial (textile dyes) WW and for harnessing renewable energy. This basic concept entails parallel or sequential modes of treating two chemically different WW *i.e.*, domestic and industrial in the same system. In this case, contemporary advancement in MFC/MEC (METs) based systems towards Microbial-Electro-Fenton Technology (MEFT) revealed a substantial emerging scope and opportunity. Principally the said technology is based upon previously established anaerobic digestion and electro-chemical (photo/UV/Fenton) processes in the disciplines of microbial biotechnology and electro-chemistry. It holds an added advantage to all previously establish technologies in terms of treatment and energy efficiency, minimal toxicity and sludge waste, and environmental sustainable. This review typically described different dyes and their ultimate fate in environment and recently developed hierarchy of MEFS. It revealed detail mechanisms and degradation rate of dyes typically in cathodic Fenton system under batch and continuous modes of different MEF reactors. Moreover, it described cost-effectiveness of the said technology in terms of energy budget (production and consumption), and the limitations related to reactor fabrication cost and design for future upgradation to large scale application.

## Abstract 2

Schematic diagram depicting catalysis of dyes and organic compounds in MXC based MEFS.

## Graphical abstract

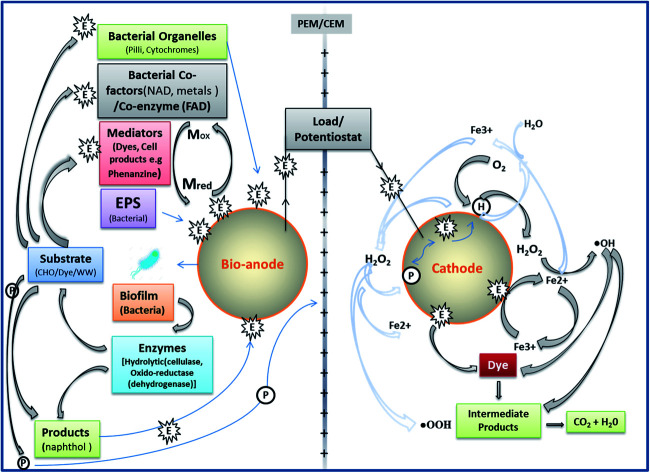

## 1 Introduction

Textiles are included amongst the most pollution causing industrial sectors into air, water and terrestrial environments. Persistent organic pollutants (POPs) such as dyes and pigments are extensively manufactured and discharged (5–1500 mg L^−1^) in textile industry effluents. Other industries including leather, foods, beverages, pharmaceuticals, cosmetics, paper and pulp are also releasing significant amounts of such pollutants in their effluent.^1,2^

Developing countries are mostly devoid of any domestic or industrial wastewater treatment facilities, while those operating are often ineffective for refractory chemicals.^3^ Moreover, power shortages and cost of treatment facilities are one of the major drawbacks. Considering these facts the wastewater generally originating from industrial units is released in existing water channels (streams and rivers) after primary settlement tanks. The refractory contaminants stay in waster channels or settle in soil and sediments depending upon their half-lives, causing enormous issue to freshwater ecology.

Dyes are highly diverse (∼100 000 types)^4^ xenobiotic and stable under ambient conditions of light and oxygen in natural ecosystems and conventional treatment plants. Such pollutants, due to their strong structural integrity and reactive nature, impose a varying degree of toxicity and mutagenicity to living systems, and create long term environmental issues.^5^ Physically, color due to dyes is aesthetically objectionable in water and more specifically red and purple as being unnatural create more concerns compared to blue, green and brown that are normally natural colors.^6^ Moreover, color prevents sunlight penetration in water,^7^ thereby limits gross ecological productivity (photosynthesis) of aquatic environment. Under these conditions, the natural processes can no longer compensate the bacterial consumption or decomposition of such and other contaminants resulting a stagnant aquatic environment.^8^ Chemically, these compounds affect the water chemistry and associated quality due to increasing load of pollutants indicators such as TDS, COD and BOD. Depleting oxygenic levels causes oxygenic stress that limits a great deal of respiratory physiology in living organisms. Whereas high levels of TDS results creates electric conductivity level that may add further toxicity. Such factors ultimately lead toward death and decay of aquatic flora and fauna.^9^

Discharge of untreated dyes containing wastewater (WW) is highly objectionable and extensive treatments are required for environmental health and sustainability. Worldwide, various traditional and advance physico-chemical treatments^10^ methods including; adsorption,^11^ ozonation, photo-catalysis (UV + H_2_O_2_), coagulation, electrochemical oxidation, and filtration have been used for textile dyes and WW from industries. Coagulation and adsorption have been the simplest and commonly used methods.^11^ Irradiation is very efficient but unsuccessful against photo stable dyes.^12,13^ Membrane filtration is highly proficient though need sophisticated nano or ultrafiltration membranes.^12^ Chemical or electro-chemical methods including ozonation Fenton process or Electro-Fenton process (EF), and advance oxidation process (AOPs) showed great prospects for degradation of both soluble and insoluble dyes, however, these treatment are either costly or facing issues of sludge waste management^12,14–18^. Moreover, both chemical and physical methods have been reported for the removal of only 20–30% dyes from WW.^19^ Biological wastewater treatments include an array of living organisms (bacteria, fungi and plants) integrated into different suspended and attached growth reactors. They are considered ultimate due to diverse physiological capabilities of organisms to be operationalized both under aerobic and anaerobic conditions for complete decomposition of dyes. Still they are slow to evolve and functionalize until given additional technical or operational support.^20,21^ Smart application of biological process may also lead to re-innovating useful products from organic waste (renewable energy & industrial products). Moreover, they are environmental friendly, cost-effective and highly sustainable.

In the last two decades considerable advancements have been made on multifaceted microbial electrochemical technologies (METs). Typically, microbial fuel cell (MFC) and microbial electrochemical cell (MEC) have been mostly developed for treatment of variety of pollutants linked to domestic and industrial sectors.^9,22,23^ METs principally work on the anaerobic bio-catalysis of contaminants for harnessing renewable energy [electrical^24^ or gaseous energy (H_2_)].^7^ Recently, advancement on MXCs as Micro (Bio-)-electro-Fenton process (MEFP) is taking lead relying on the previously established WWT technologies typically including EFP, AOP, biological processes and MES.^15,18^ MEFS using MFC or MECs have gone through a series of innovations and advancements.^25–27^ In 2000, Lin and Chang, reported a combine sequential approach of treating landfill leachate and refractory compounds using EF and biological processes^28^ Later, in 2009, the 1st MEFS was developed for aerobically stable pollutant like dyes and it further expanded the scope of such innovative technologies.^27,29–33^ As compared to traditional EF technologies, the MEF technology is more efficiency and cost-effective (approx. 10 fold) for refractory compounds in WWT.^21^ Besides it creates almost negligible amount of sludge. This technology offer superior possibilities of treating persistent organic pollutants (POPs) of xenobiotic nature in WW^34,35^ including dyes,^32,36,37^ pesticides, dioxins, personal care products and pharmaceuticals^24,38,39^ lignin (paper and pulp), bagasse and molasses from different industries. Besides, it is equally effective for municipal and agriculture waste treatment under varying physicochemical loads.^32,40^

A basic MEFS include a similar METs set up^21^ for anaerobic degradation of biodegradable compounds in anodic chamber, however, it is also supplemented with a Fenton Process (FP) for simultaneous treatment of refractory compounds (dyes, pesticides or aromatic compounds) in a non-selective manner in cathodic chamber.^34^ Cathodic oxidation of contaminants involves oxygen species as hydroxyl radicals that are highly reactive and generated through catalysis of H_2_O_2_ by Fe^2+^.^10,41,42^ By Fenton's reagent, oxidation of organic compounds is rapid and exothermic process that helps almost complete mineralization of contaminants into inorganics *i.e.*, carbon dioxide and water.^29^ Additionally reports have also suggested possibilities of coupling photo-catalytic (UV/light) oxidation along with typical FP to enhance the overall efficacy of MEFS.^21^ An added advantage of this innovative technology is treatment of two different wastewaters (pollutants) in the same reactor. In the last few years, further improvement in architecture and scope of MEFS helped development of new reactors that are yet to be operationalized at pilot and large scales.

## 2 Dyes and pigments; characteristics, source and environmental fate

### 2.1 Characteristics of dyes and pigments

Worldwide, an annual production of various dyes and pigments is about 700 000 tons.^4^ Generally dyes and pigments are made up aromatic backbone structure of benzene with alternating double C bonds (delocalized electrons pairs) that helps in transmission and reflectance of natural white light resulting in creation of different hues.^1^ From an industrial perspective, successful dyes have a strong structural integrity and high water solubility for possible sustainable application in different products.^8^ On the other hand, pigments are made hydrophobic with large molecular structures and intermolecular attractions due to hydrogen bonding (N–H and C=O groups).^43^ Globally, 40% of colorants used comprise chlorine that is organically-bound.^6^ With their widespread applications and complicated chemistry, the utilization of colorants in textile industry could also be a major cause of dioxins and their precursors that typically relates to POPs. Being the most toxic compound, dioxins are associated to halogens homologues. The recalcitrant nature of dyes and pigments is typically associated with their benzoic structure that makes them highly objectionable as they avoid natural process of mineralization under normal oxygenic environment^44^ thereby creating environmental issues of low biotic productivity. The reactive nature of such compounds due to presence (*e.g.*) chloro, nitro, and sulphonic groups make them toxic and mutagenic at varying degrees at different trophic levels.

The basic structure of dyes and related compounds comprises a chromophore group represented by “the azo group (–N=N), ethylene group (=C=C=), methine group (–CH=), carbonyl group (=C=O), carbon-nitrogen (=C=NH; –CH=N–), carbon-sulphur (=C=S; ≡CS–S–C≡), nitro (–NO_2_; –NO–OH), nitrozo (–N=O; =N–OH) or chinoid groups. The auxochrome groups are ionizable groups that confer to the dyes the binding capacity onto the textile or any material. The usual auxochrome groups are: -NH2 (amino), –COOH (carboxyl), –SO_3_H (sulphonate) and –OH (hydroxyl)”.^45^ Overall, the major classes include; azo or anthraquinone accounting for 65–75% in textile dyes. An about 2/3 of them are azoic in nature. The precursor compounds or intermediates are anline, chloroanilines, naphthylamines, methylanilines, benzidines, phenylendiamines, and others.^6^ Azo dyes are characterized by the reactive groups that make covalent bonds with HO–, HN–, and HS– groups present in fibers *e.g.* cotton, silk, nylon, wool. These dyes are frequently utilized for the yellow, red and orange colors.

### 2.2 Classification of the dyes

Environmental positioning of dyes is related to; C atoms number and the aromatic structure, number and nature of substituents, and overall backbone molecular structure.^6^ Typically, classification of dyes and pigments is either based upon their chemical structure or their mode of applications to different substrate products. Structure based classification is related to the nature and chromophore groups that helps predicting dyeing and associated oxidation – reduction. There are 8 different categories of dyes based upon their chemical structures. Dyes based upon application depends on chromophore grouping that help dying based upon dye solubility in dye bath and its affinity with different fibers and nature of fixation. On solubility basis, dyes and pigments are broadly characterized as anionic, nonionic and cationic in nature.^46^ Among anionic dyes (direct, acid and reactive) reactive and acidic are most problematic due to their bright colors and water solubility in conventional wastewater treatments.^6^ Nonionic dyes such as disperse do not ionize in the aqueous system, while, cationic ones are anthraquinone, azoic, reactive and disperse dyes. Few disperse dyes are efficiently bioaccumulate and their azo and nitro groups are reductively cleaved (reduced) in soil sediments to toxic aromatic amines.^6^ On this basis, they are 9 different types partially distributed in the two categories of water soluble and insoluble. Some basic details on classification of dyes is mentioned in Table 1.

**Table 1:** Classification and properties of Dyes

Category (structure and mode of application)	Sub-category (type/nature)	Chromophore	Auxochrome	Properties/examples	Discharge rate (%)	Fabric type	Structure and chemical formula	References
Chemical structure	Azo	–N=N-	NH_2_, OH, SO_3_, Cl	70% of synthetic dyes, mon, di, tri azo. Yellow reactive 4, black reactive 5	5–10	Cellulose	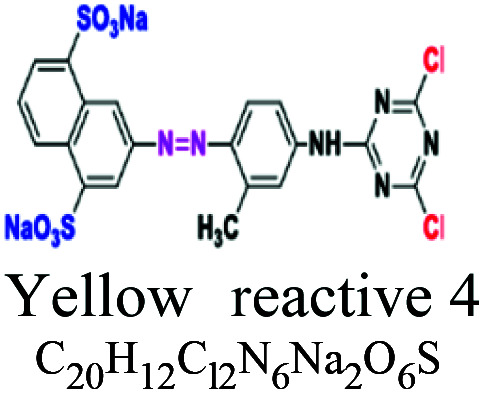	47
	Anthraquinone	=C=O. and =C=C=, forming an anthraquinone complex (quinone nucleus)	NH_2_, OH, SO_3_	Most important after azo, derived from anthracene. *e.g.*; acid blue 62, reactive blue 19	2–10		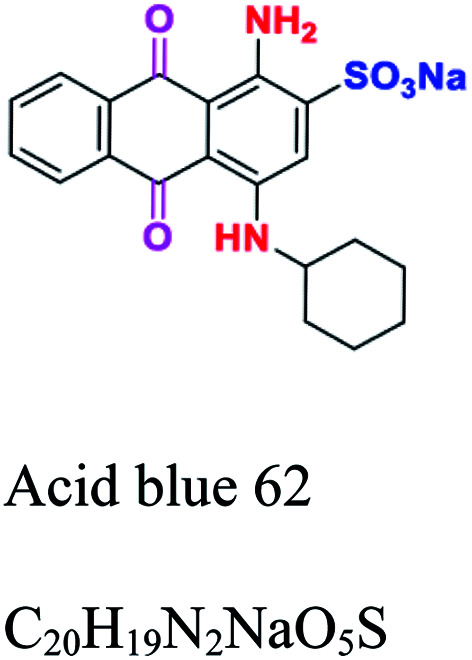	46
	Indigo	C=O, NH_2_, C=C, C–O, C=N, C–C	SO_3_, NH_2_, C=O	Derived from indigo. *e.g.*; blue acid 74, indigo blue			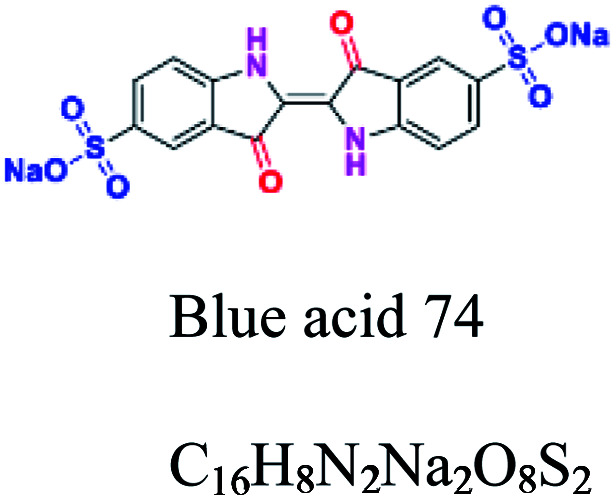	48
	Xanthene	Xanthylium or di-benzo-g-pyran nucleus	Amino or hydroxyl, COOH	Intense fluorescence, used as markers/tracers in maritime accidents or underground river. *e.g.*; fluorescein, eosin, erythrosine			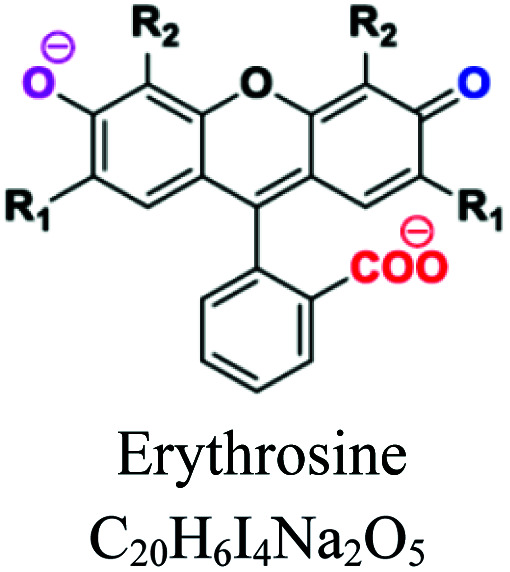	49
	Phthalocyanine	Phythalocyanine nucleus	SO_3_	Metal complex (Cu, Ni, Co, Pt *e.g.*; copper phthalocyanine. *e.g.*; pigment blue 15/3, nickel(ii) tetrasulfonic acid			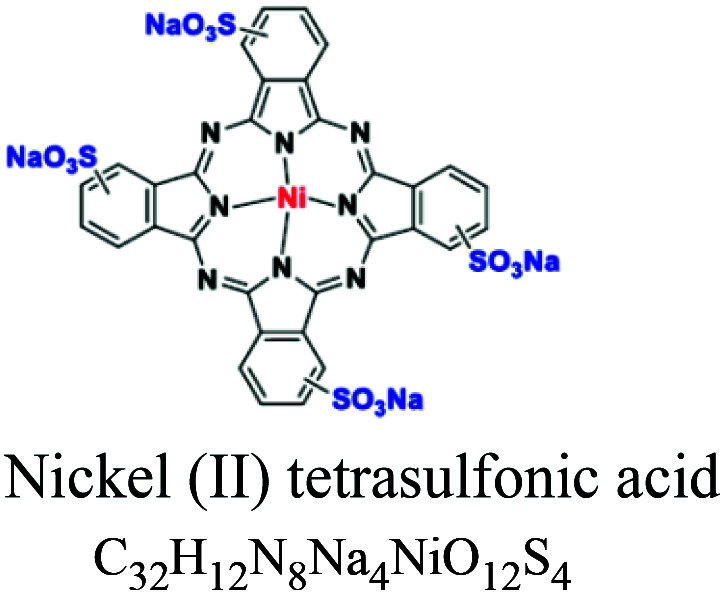	32
	Nitrated & nitrosated	O–NH=O, O–NH=O, phenol, nitro	Nitro (–NO_2_), OH, NH_2_, CH_3_NH	Limited number, older *e.g.*; picric acid (2, 4, 6-trinitrophenol), 2-nitrophenol, 2-amino-4-nitrophenol			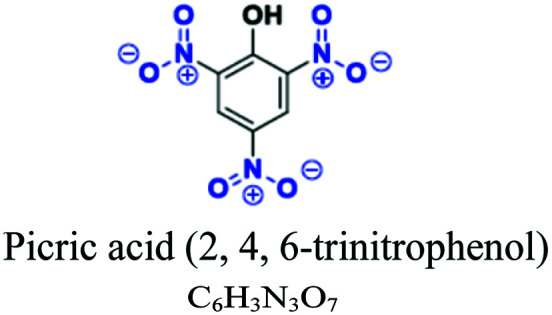	50
	Diphenylmethane & triphenylmethane		CH_3_, NH, SO_3_	Oldest, derivative of auramine and triphenylmethane *e.g.*; fuchsin and malachite green			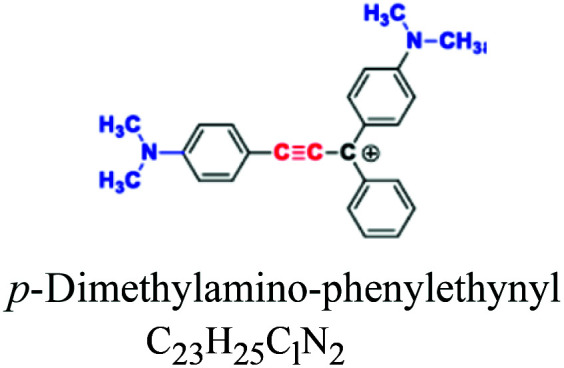	51
	Polymethinic	=HC–HC=CH–CH=	OH, SO_3_^−^, CH_3_, HN–N–CH_3_	Also called cyanines, *e.g.*; basic yellow 28, polymethine dye 2630			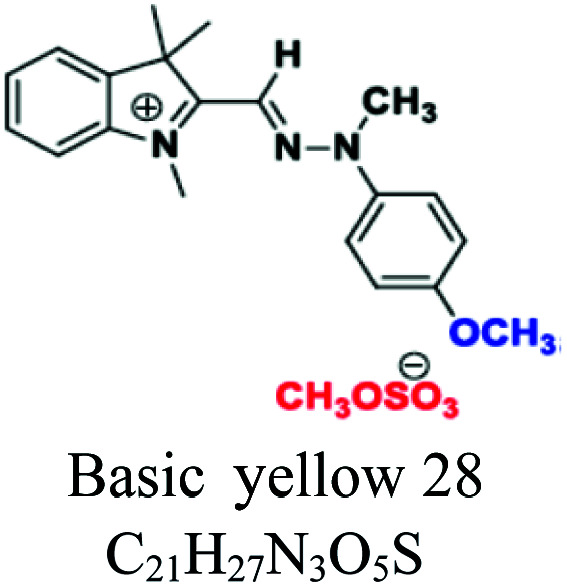	52

**Application**								
H_2_O soluble	Acid or anionic	Azo, anthraquinone or triaryl	SO_3_^−^, NH_2_, OH	For wool, polyamide, silk and acrylic, *e.g.*; red acid 27, acid blue 90, acid blue 74	7–20	Wool & nylon	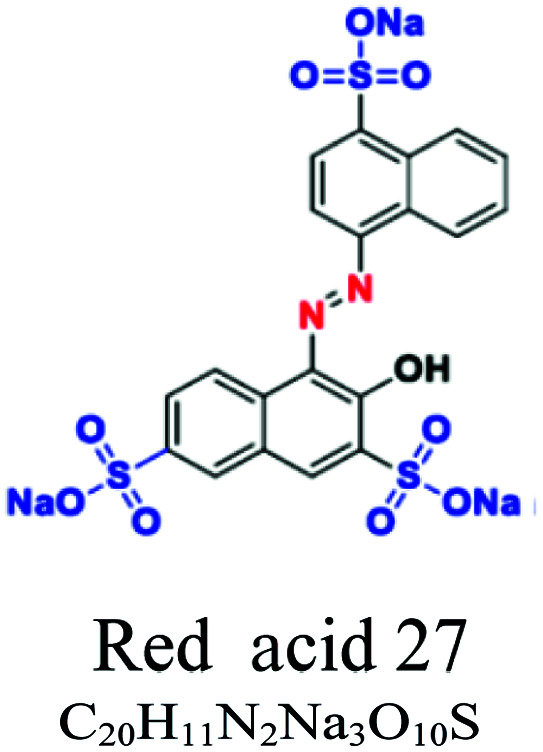	53
	Basic or cationic	Diaryl, triaryl, anthraquinone, azo, phthalocyanine	NH_2_	*e.g.*; basic blue 9, basic yellow 37, blue nile	2–3	Acrylic	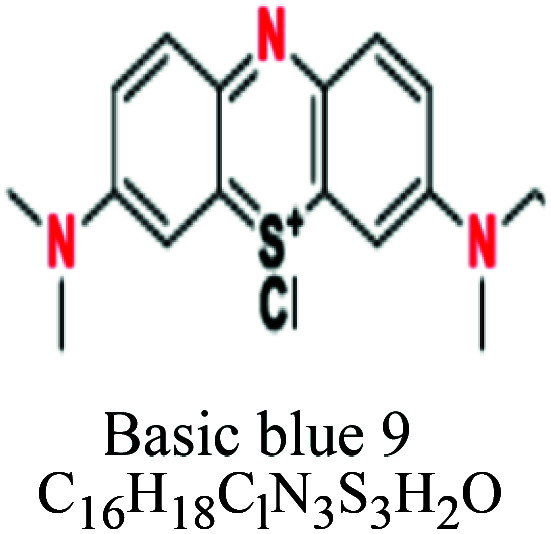	54
	Metalliferous	Azo, phthalocyanine		Acidic dye with metals (Cr, Cu, Ni and Co)	2–10	Leather, finishing, stationery, printing, inks, inks, coloring for metals, plastic		32
	Reactive	Azo, anthraquinone, and phthalocyanine	SO_3_, NH_2_	*e.g.*; reactive red 198	10–50	Wool and nylon	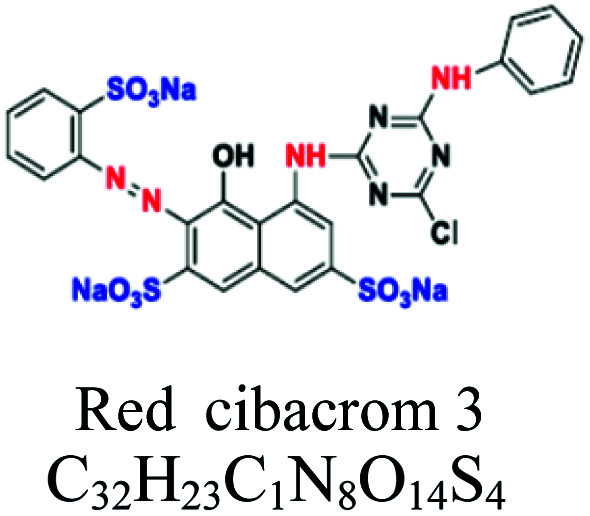	55
	Direct or substantive	Azo, phthalocyanin	NH_2_, SO_3_, OH	Large molecules with positive and negative charges, affinity to cellulose, wide variety, easy to apply and low cost, *e.g.*; direct blue 1	5–30	Cellulose	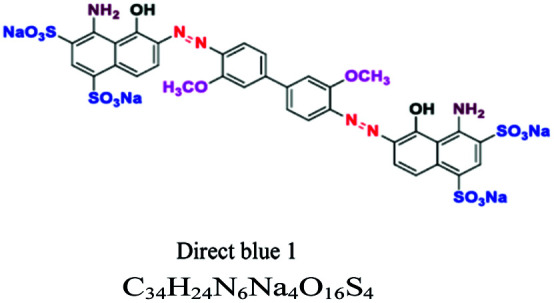	8
H_2_O insoluble	Vat dyes	Anthraquionone	NH_2_, C=O, CH_3_	Good resistant to degradation (soap and sunlight), good affinity to cotton, linen, wool, silk, rayon, use for dyeing jeans. *e.g.*; indigo (vat blue 1), blue indanthrene RS (vat blue 4, vat green 1	5–20	Cellulose	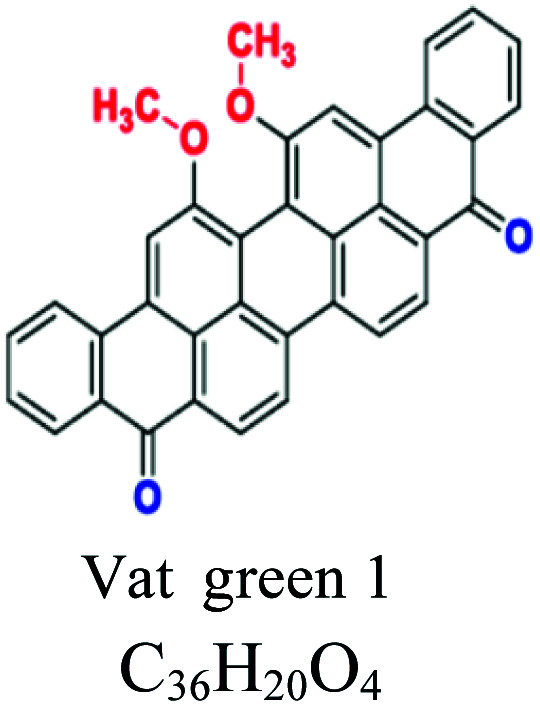	56
	Sulfur	Thiazoles, thiazone, thianthrene, and phenothiazonethioanthrone	NH_2_, OH, SO_3_	Like vat dyes, complex high MW, used for cotton, dark shades, *e.g.*; sulfur black, sulfur blue 15	30–40	Cellulose	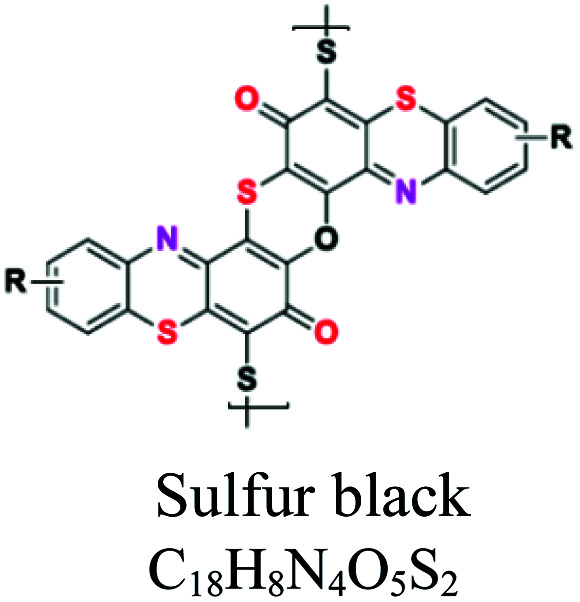	8
	Disperse or dispersible	Anthraquinone	NH_2_, OH	Plastosolubles, stable at high tempeture, used in polyester and polyamide, *e.g.*; blue disperse 7, red disperse 60	2–20	Synthetic	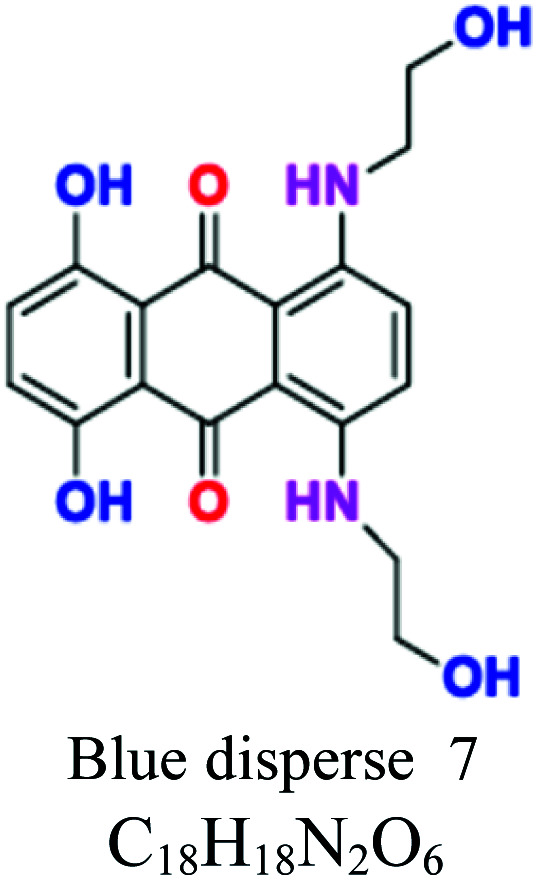	8
	Pigments			Organic (benzoic), inorganic (metals; Ti, Zn, Ba, Pb, Fe, Mo, antimony, zirconium Ca, Al, Mg, Cd, Cr), kept in suspension tanks to dispersants, film formation by heating, used in printing		Paint, ink, plastic, fabric, cosmetics, food, and other materials		43

### 2.3 Source and environmental fate of dyes and pigment

Industrial units including textile, pulp/paper, leather tanning, dyestuff manufacturing, pharmaceutical and kraft bleaching industries have been reported as major point sources contaminating dyestuffs in water sources.^6^ Typically dyes are released in water ways from dyebath (dyeing unit) and dyes manufacturing units of industries.^57^ The annual estimated load of pollutants in textile wastewater varies from 200 000–250 000 t salts. The exhaustion rate of dyes and pigments from dyebath unit is 10–60% comprising 10–50% reactive dyes, 5–20% vat dyes, 5–30% direct dyes and 10–40% sulphur dyes and that is 280 000 tons per year.^1^ Release of raw or partially treated effluents adds a huge load of TOC, nitrites, nitrates, phosphates and heavy metal ions (zinc, iron, mercury, lead, chromium, cobalt and copper) thereby also creating an issue of eutrophication. Typically, textile dyes are highly relevant in ecological perspective. Among them, azo are taken as model dyes due to their large production, consumption (60–70%) and discharge in the hydrosphere. Effluent of leather dyeing may contain greater than 250 ppm dyes causing an about 7000 ppm COD load. It is highly acidic (pH 4.5 approx)^58^ although alkaline in the case of the textile industry. Effluents from these industries are also source of dioxins because of the presence of dioxazin and anthraquinone dyes and pigments made from chloranil (tetrachloro benzoquinones) and pentachlorophenol. Dioxins are generally released during manufacturing and treatment of dyes by photolysis/UV.^59^

Long time persistence (*i.e.* half-life = several years/2 − 13 years) of dyes accumulate them in abiotic sediments or living or dead biomasses particularly in fish and other aquatic forms of life. Mostly they are soluble still they tend to biosorb or absorb and accumulation in suspended and settled abiotic and abiotic components of water bodies. With solubility greater than 2000 mg L^−1^ in water, bioaccumulation is not estimated for dyes. Adsorption is high in basic and direct dyes while range is from high to medium in disperse dyes, and it depends on degree of sulphonation or ease of hydrolysis. In group 1, ability to adsorb on the biomass and water solubility is increased due to higher levels of sulphonation. In Group 2, dyes are also greatly sulphonated but indorsed good adsorption performance on sludge moderately. Additional information in bio-elimination of different reactive dyes stated that disazos, anthraquinones, triphendioxazines and phthalocyanines are usually better adsorbed than monoazos. It is crucial to highlight that toxic compounds *e.g.* benzidine, aromatic amines and its derivatives can be produced in environment *via* transformation of textile dye precursors such as by reduction or hydrolysis of azo dyes. Moreover, sequential bioaccumulation in entire food chain^60^ and later biomagnified at higher trophic levels cause lethal effects.

As dyes are photo-chemically stable under ambient environmental temperature, so, they normal create problem in conventional industrial and residential treatments systems.^61^ Dyes are highly polar (log *K*_ow_ up to 3) and shows recalcitrance also depending on environmental variables such as redox milieu or pH. A compound like aniline (1^st^ synthetic known dye) has been found easily degradable under aerobic condition; however, it is highly stable under anoxic condition.^62^ The recalcitrant and xenobiotic nature of dyes usually influence the structure and function of aquatic ecology.^57^ Additionally these compounds also inflict a negative effect on microbial communities of the soil and germination of plants. Typically, living species at the higher trophic levels of the food web are exposed to a thousand times higher concentration of toxicants than those at lower levels.^8^ Ecological and Toxicological Association of Dyes (ETAD) reported that 98% of dyes have LC50 value higher than 1 mg L^−1^ for fishes and 59% of dyes have LC50 greater than 100 mg L^−1^ and 28% higher than 500 mg L^−1^. LD_50_ reported for most of the azo dyes varies between 100–2000 mg kg^−1^ body weight.^62^ They affect physiological status of biotic community and in England, it was reported that inhibition of respiration rate in sewage bacteria is caused by 18% of 200 dyes.

Dyes generally composed of benzene moieties that makes them recalcitrant (xenobiotic nature) toxigenic and mutagenic.^63^ Additionally, certain functional groups such as halogens (chloride), amide, amino, sulphides and metals may add further toxicity and mutagenicity at different trophic levels.^61,64^ They may also carry precursors of toxic compounds like dioxins that as by-products produced during the synthesis of chlorobenzenes, chlorophenols, polyvinyl chloride, chlorobiphenyls, pigments, dyes, and printing inks. They can even be toxic at 1 mg L^−1^ concentration in effluents so that they are related to environmental deterioration and various diseases in living beings.^61,65^ Despite recalcitrance they are potentially degradable or transformable in anoxic environmental condition and soil sediments, releasing sometime toxic compounds. Acute toxicity to textile dyes relates to ingestion and inhalation,^66^ causing bladder cancer,^67^ dermatitis, nervous disorders^65^ impaired enzymatic activities,^68^ eliciting irritations to the eyes and skin.^67^ The personnel handling and producing reactive dyes may have contact rhinitis, allergic conjunctivitis, dermatitis, asthma or other allergic reactions.^67,69^

This persistence nature of dyes has also been closely related to their reactivity. It increases with the number and types of electron donating substituents typically halogens, nitro, sulphonic and alkyl groups.^8^ Besides, substituents in *ortho* and *para* position further upsurge the carcinogenic potential. Essentially, the toxicity lessened by the protonation of aminic groups.^6^ Highly reactive nature of dyes and aromatic products makes them potential candidates to transform biomolecules at very minor to major levels in the living bodies. Such changes in the living biomolecular structure impair the physiological status of living organism that becomes sever under hypoxic conditions in aquatic sediments or living biomass. Under extreme situations azo dyes may leads to DNA damage that can result in mutation and cancerous growth and sometime lethal effects like death. A substantial decline in tadpole survivorship was witnessed at 209 μg g^−1^ while a major upsurge in malformations at two peak concentrations was tested in sediment.^70^ Especially in the case of azo dyes, carcinogenicity can be produced by both the dye itself and its own metabolized compounds.^67^ Few well-known azo dyes *i.e.* azodisalicylate, precursor for 4-phenylenediamine; Direct Black 38, precursor for benzidine and their derivatives are benzidine and its derivatives, and also higher number of anilines *i.e.* 4-chloroaniline, 2-nitroaniline, 4-phenylenediamine, 4,4′-dimethylendianiline, dimethylamines, nitrosamines *etc.* causing cancer in humans and animals.

At genotoxicity level, large concentrations of dye (DY7) in sediment prompted cellular stress-related gene transcription and influenced the genes associated with chromosome condensation, necrotic cell death, and mRNA processing.^70,71^ Dyes may^67^ create chromosomal aberrations other times mutagenic.^69^ Dyes like Azure B found to be affecting helical structure of DNA^67,72^ and duplex RNA.^73^ Whereas at cytotoxic level, they inhibition enzymes (monoamine oxidase A) (MAO-A),^74^ involved in nervous system^75^ related to human behavior.^76^ Similarly, inhibition of glutathione reductase^77^ disturbed redox homeostasis cellular.^78^ Disperse red 1 dye has been reported exhibiting mutagenic potential^79^ on human lymphocyte and hepatoma (HepG2) cells, hepatocyte imitative cells,^80^ causing occurrence of micronuclei,^81^ indicated mutagenicity at chromosome level.^82^ Besides, it caused DNA adducts^83^ a key cancer causing event in *Salmonella* spp.^84^ Similarly, disperse orange 1 exhibited mutagenicity^79^ causing DNA damage-a base pair substitution and frameshift mutations in *Salmonella* spp. Moreover, it caused cytotoxicity with apoptosis in HepG2 cells. Another dye, Sudan I (solvent yellow 14),^85^ although illegal, used as a food supplement in paprika, has enzymatically transformed into carcinogenic aromatic amines by intestinal microbiota. In rats, the presence of Sudan I dye is confirmed by neoplastic liver nodules. Basic red 9 a commonly used dye in textile, leather, paper and ink industries^86^ indicated carcinogenic potential in human and environmental^87^ after partially degradation into carcinogenic aromatic amines in anaerobic conditions. Disposal of such compounds in water bodies showed potential for allergic reactions, skin irritation, mutations and cancer. Besides they comprise local sarcomas and tumors in the liver, bladder,^88^ mammary glands and hematopoietic system. Crystal violet, a cationic dye (triphenylmethane group),^89^ caused mitotic dysfunction and suspension at metaphases^90^ and inducing disruption of chromosomes in Chinese hamster ovules. Crystal violet has also been reported promoting fish tumors, and hepatocarcinoma, reticular cell sarcoma in vagina, uterus, ovary and bladder, hardened gland adenoma and ovarian atrophy in rats. Besides, it caused cystitis, irritation of the skin and digestive tract, respiratory and renal failure.^90^

## 3 Degradation mechanism of dyes and organic compounds in MEFS

**Fig. 1:**
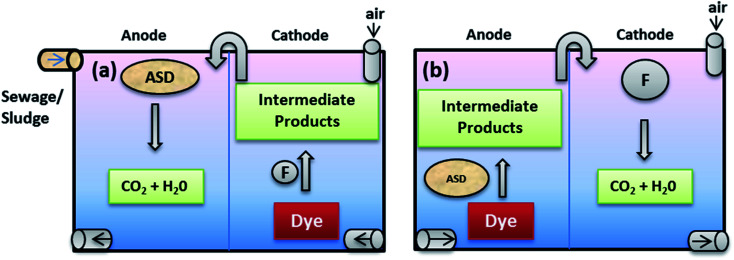
Model concept of MXCs based MEFS: sequential batch or continuous modes; (a) aerobic degradation of dyes in cathode through Fenton reaction (FR) followed by anaerobic mineralization of dyes products and sewage/sludge in anode, (b) anaerobic degradation of dyes and sewage/sludge in anode followed by aerobic mineralization of products of dyes and sewage/sludge in cathode through FR (ASD = anaerobic sewage sludge digestion; F = Fenton agent ˙OH).

MEFS is an integration of biological (microbial) degradation with that of electrochemical process *i.e.* Fenton process, for efficient removal of pollutants in less time. In a typical Microbial-electro-Fenton processes using a two chamber MFC, electrons originating from anaerobic bio-catalysis (oxidation) of organics and inorganics follows an anodic pathway reach the cathode to combine to reduce (2 electron) O_2_ to form H_2_O_2_ (79 to 196 mg L^−1^) with simultaneous electricity production in 1^st^ stage. Then it follows the production of highly reactive (oxidative) OH radical through chemical catalysis (Fe) of H_2_O_2_ in the 2^nd^ stage. The enhanced oxidation of the target compounds such as dyes through hydroxyl radical is the final 3^rd^ stage. MEC, an extended version of MFCs, requires a minor input of electricity (0.2–0.8 V) in order to accelerate overall performance and H_2_O_2_ production (1300–1447 mg L^−1^). A part of free energy formed is taken by bacteria for catabolic activities and rest can be employed to produce electricity for maintainable operations of the system.^91–94^ Such systems can equally be good to treat two different dyes and their byproducts at 1^st^ and 3^rd^ operational stages simultaneously. Another good possibility can be sequential treatment (back and forth) of same refractory dyes in the two chambers of reactor in order to achieve complete mineralization as shown in Fig. 1. Beside these systems creates a workable electric potential to facilitate reactor performance and make them cost-effective and sustainable. A biological catalyst is either an enzyme or bacteria immobilized on the anodic reactions. Although the cathodic reactions may involve a biological or chemical catalyst in a typical MFC.^40,95,96^ For advanced oxidation of refractory compounds in cathodic chamber, a chemical catalyst like Fe^2+^ is utilized to generate OH radical from H_2_O_2_.

Microbial electrochemical systems depend on diverse bacterial communities that encompasses electricigens or ARB. In the anode compartment, a mutualistic activities of several anaerobic microorganisms (fermentative, syntrophic, acetogenic, and methanogenic) consequent in the degradation of complicated organic substances into simple and stabilized compounds, primarily, methane and CO_2_. Initially such systems often operated with glucose and acetate as a fermentable substrate to upkeep the growth of electricigens. The fermenters and other syntrophic bacteria perform hydrolytic monomerization and fermentation of complicated substrates^97,98^ from food, beverages, pulp and paper, leather, pharmaceutical and residential wastewater. The enrichment and catalysis role of fermenters helps improved oxidation rates of monomers by electricigens in the secondary stage.^99^ It also work to ease the role of hydrogen scavengers.^100^ Generally, anaerobic digestion (AD) involves liquefaction or hydrolysis of insoluble compounds,^101^ followed by acidogenesis/acetogenesis and methanogenesis.^102^ Bacteria like *Streptococcus* and *Enterobacterium* release extracellular hydrolytic enzymes *e.g.* cellobiase, xylanase, cellulase, amylase, protease, and lipase to degrade biopolymers^103^ into simple monomers and dimers *i.e.* monosaccharides, fatty acids and amino acids.^104–107^ In the 2^nd^ stage, monomeric products are then transformed into “short-chain organic acids (formic, acetic, propionic, butyric, and pentanoic) alcohols (methanol, ethanol), aldehydes, carbon dioxide, and hydrogen by acidifying bacteria including *Pseudomonas*, *Bacillus*, *Clostridium*, *Micrococcus*, or *Flavobacterium*”.^105,108,109^ In the 3^rd^ stage, acetogens including *Syntrophomonas* and Syntro-phobacter transform the acid phase products into acetates and hydrogen that could be utilized by methanogens^110^ in syntrophic relation with acetogenic bacteria.^110,111^*Methanosarcina* and *Methanosaeta*, acetogenic methanogens, generate methane that accounts an about 2/3 in AD in the 4rth stage.^112,113^ Methylated compounds and Carbon dioxide are the alternate substrates for production of methane by *Methanosarcina*, *Methanothermobacter*, and hydrogenotrophic methanogens. Whereas, methylated compounds are appropriate substrates for *Methanosphaera*, *Methanosarcina* and methylotrophic methanogens.^113^ Overall, biogas production during AD comprises 65–70% methane and 30–35% carbon dioxide.^114^

Typically MEFS based reactors demonstrated enrichment of diverse ABR bacterial community in anodic biofilms.^115^*Proteobacteria* has been reported most abundant phylum under these setups due to its versatile abilities for aromatic compounds (azo dyes) biodegradation, electricity generation, and fermentation.^116^ Functionality based analysis showed chemo-heterotrophy the predominant followed by fermentation (6.6 ± 1.6%) and nitrate reduction (7.4 ± 2.3%). Two distinctive electricity producing bacteria *i.e. Shewanella* and *Geobacter*,^117^ were anticipated to be the key players in the improvement of iron respiration.^118,119^ Other bacteria with selective relevant function include *Serratia*, *Stenotrophomonas* for EET, *Stenotrophomonas Dysgonomonas*, *Brevundimonas*, and *Achromobacter* for reduction of azo-dye or degradation of aromatic compounds.^120–122^ The relative abundances of fermenters *Acinetobacter*, *Cloacibacillus* and methanogens *Methanobacterium* were lowered under MES systems.^123–125^

Compound like azo dyes from dyeing and production sectors of industries account for an about 60% of the total known dyes. Usually they are taken as model compounds to be treated in any biological, physical or chemical treatment. Azo dyes have shown partial to complete mineralization in most of the MEFS.^20^ Still, most of the previous studies have reported microbial-electro-Fenton process achieving complete degradation of azo dyes including amaranth, orange G, methylene blue, crystal violet, Lissamine green B, and orange II by integrating effective electro-Fenton reaction in the cathode chamber.^126–128^ Generally, for azo dyes removal two different types of MEFS such as MFC or MEC and their modified version have been used. Azo dyes due to their charged large structures are expected to be reduced either extracellularly or with membrane bounded enzymes. Under anaerobic condition azo dyes cleavage at N=N to produce colorless aromatic amines. Anodic catalysis in MFC using microbial consortia broadens the horizon of substrates utilization due to diverse microbial metabolic apparatus in terms of enzymes, coenzymes and cofactors this helps improves the energy efficiency and less toxicity in the system.^129–131^

During anodic biocatalysis of dyes the role of oxidoreductases (dehydrogenases (alcohol, formaldehyde, formate, fructose) oxidases, oxido-reductases), cofactors and coenzymes “(nicotinamide adenine dinucleotide (NAD^+^), nicotinamide adenine dinucleotide phosphate (NADP^+^), pyrroloquinoline quinine (PQQ), Flavin adenine dinucleotide (FAD) have always been vital (Fig. 3). In case of FAD cofactor dependent oxidoreductases, three FAD-dependent glucose dehydrogenase (FAD-GDH), cellobiose dehydrogenase (CDH) and fructose dehydrogenase (FDH) have been extensively studied”.^132^ Additionally, the role of natural and artificial mediators (methylene green, methylene blue, neutral red,^133^ phenazines, methylene blue, alizarin yellow, methyl violet, thionine, Prussian blue, azure A, toluidine blue and azure C) that reacts with reduced enzymes or coenzyme in order to transfer electrons to electrode is highly effective.^134–137^ Moreover, the degradation products of dye (AO7) such as naphthol have been reported as an alternative for electron mediation.^138^ On the other hand, the electrode can also reduce enzyme thereby enzymatic reduction of substrate *i.e.* dye (bioelectrocatalytic reduction). Direct enzymatic reduction of electrode proved to be more effective, however,^139,140^ if cofactors are involved then they should not be 20 Å (2 nm) away from electrode surface.^141,142^

Metals and their alloys have also been reported playing a major role as catalyst due to their different oxidation state in Microbial-electrochemical Fenton system. These metals include Pt, Au, Ni, Co^143^ Fe and Cr. Ti, V, Cr, Mg, Mo, and Re.^144^ However, they are sometime expansive,^145^ susceptible to adsorption by the impurities^144,146^ and show limited substrate specificities. “Commonly occurring metallocofactor motifs of metalloenzymes are: haem centres, Fe–S clusters ([Fe–S]), Fe centres, Cu centres, Mo centres (Mo-cofactors, Moco) and W, including various iterations and combinations. In many cases, only a single catalytic redox cofactor is found, although others can be found to be involved in internal ET (commonly the case of [4Fe–4S] containing proteins). Frequently, oxidoreductases that comprise non-metallocofactors (FAD) and PQQ-dependent enzymes also utilize metallocofactors to transfer electrons to and from their redox partners, or in other cases electrode surfaces. Additionally, metalloenzymes have been known to function as a transmembrane enzyme that functions on either side of a membrane or the enzyme contains a deeply hidden catalytic redox cofactor. One of the most fascinating examples of such an internal ET pathway can be found in prokaryotic nitrate reductase (Nar NR), such as that in *Escherichia coli* (NarGHI NR)”.^147^ Modern studies have reported that Mtr respiratory pathway in *S. oneidensis* MR1 involve OmcA/MtrC playing role of “azo reductase”.^148,149^ Where flavins have been reported to assist and improve the decolorization process.^150^ Besides, removal of dye (AO-7) was largely associated with bio-reduction due to dehydrogenase activity (DHA) rather than electrochemical reduction.^119^ Greater DHA activity reflected greater transfer activity of intracellular electron *e.g.*, AO-7 reduction by anodic bacteria, but some of them showed EET capability.^151^

Different bioanodic degradation pathways and byproduct have been reported in case of dyes. Fig. 3 reveals a typical anaerobic degradation an azo dye methyl orange 7 (MO7) that possible occurs in anodic compartment generating by products including *N*,*N* dimethyl-benzene 1,4 diamine and sulfanilic acid. The reaction initiated through intra-cellular dehydrogenases producing NADH from oxidation of reduced C compounds. Azoreductase then reduced the azo bond using reduced mediator from NADH generating primary extra-cellular degradation product of dye. In a typical MFC anode using *S. oneidensis*, a symmetrically cleavage pathway of AO7 was predicted after reduction of the azo bond in the creation of aminobenzene sulfonic acid (sulfanilic acid), 1-amino-2-naphthalenol (1-A-2-N), and 2-amino-1-naphthalenol.^152^ Previously, the existence of aminobenzenes under anaerobic decolorization of AO7 was reported by.^153^ In case of mordant azo dyes salicylic acid derivatives were numerically predominant byproducts, whereas, *N*,*N*-dimethylaniline, sulfanilic acid and sodium sulfanilate in case of methyl orange.^154^ Likewise, microbial or electrochemical degradation of AO-7 produces 1-amino-2-naphthol (AN) and 4-aminobenzenesulfonic acid (AA) in anaerobic environments.^20^ However, mineralized of a Congo red indicated less toxic and more degradable organic byproducts such as maleic acid and malonic acid in MEF-COR system.^35^ Current density has been reported to increase with the higher concentration of azo dyes. It further helps in degradation of dyes.^155^ Beside higher current efficiency puts a low energy demand in the system, nonetheless, required extended time for achieving acceptable mineralization.^17^ Despite MFC being energy gaining rather consuming, it has CE loses due to dyes and their increasing concentrations of dyes (AO-7) and electron consumption.^119^ In some cases, degradation of azo-dyes displayed prompt electricity generation^156,157^ compared MFCs inoculated sludge. This has been linked with products of dye such as phenazine, aminophenol, and naphthol playing role as electron mediators in electricity generation.^138,158^ Mostly reports have indicated the production of toxic or mutagenic byproducts of partially degraded azo dyes in conventional anaerobic digestion (AD). Such compounds were preferred to be treated under aerobic conditions for complete mineralization. So, current approach of treating dyes in MEFS is considerably required. Sometimes, dyes or their products became toxic due to synergistic effects of several chemical or biological constituents in the effluents instead of one or certain contaminants.

**Fig. 2:**
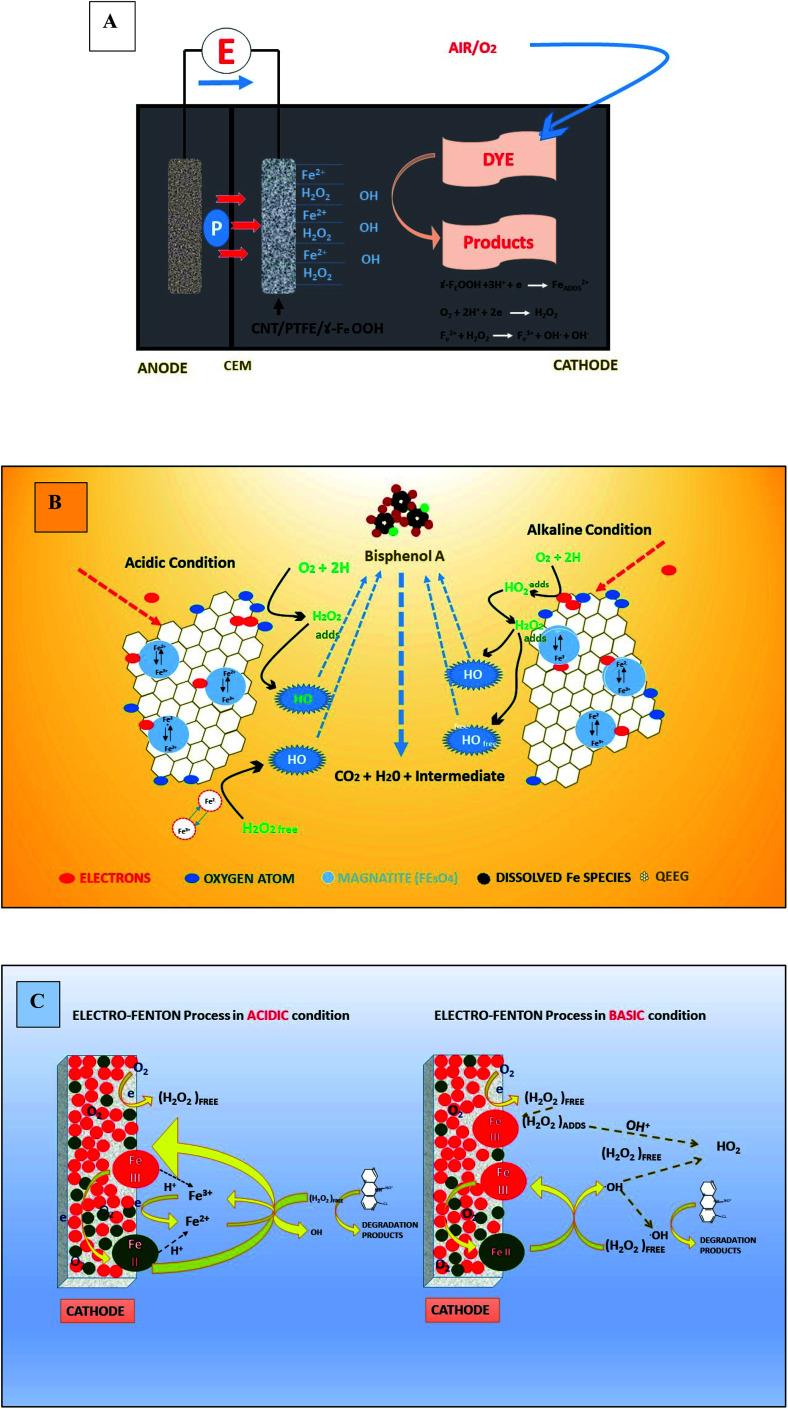
Cathodic electrochemical Fenton reaction: (A) a typical MFC; mechanisms of reactive oxygen species formation under acid and alkaline conditions (B) the graphene/Fe_3_O_4_ composite electrode (refabricated from ref. 161) (C) Fe_3_O_4_@Fe_2_O_3_/ACA cathode (refabricated from ref. 162).

Iron (F^2+^/Fe^3+^) is a key player in cathodic Fenton reaction. In a typical Fenton reaction ferrous salt (Fe^2+^ ions) in the catholytes catalyze the production of ˙OH from H_2_O_2_.^10^ Usually Fe^2+^ (Fenton) is preferred over Fe^3+^ (Fenton-like), however, a combine blend of both (*e.g.*, magnetite and ilmenite) in pure homogenous or heterogeneous and in conjugation form with other cations^59^ and composite electrodes can be effectively utilized. In case a typical Fenton reaction, powerful ˙OH is generated (*E*° = 2.80 V) (*k* = 63 M^−1^ s^−1^). Whereas in Fenton like reaction, less powerful ˙OOH radical (*E*° = 1.65 V) is released and causing a rate limiting step in hydroxyl radical production. Moreover, Fe^2+^ is constantly renewed through cathodic reduction of Fe^3+^ and though chain of other Fenton reactions.^159^ Reports have clearly validated better performance of Fenton reaction rather than Fenton-like reaction in pollutants (dyes) degradation.^23^ For optimum MEFS, factors like type and concentration of Fe catalyst and pollutant, electrodes surface area and spacing, bio-electrochemical potential and water quality are vital. It is reported that rise in Fe^2+^ concentration improved the rate of degradation of pollutants (dyes), but the rate declines above a definite value because of acceleration of parasitic reactions between Fe^2+^ and hydroxyl radical thereby loss of the system efficiency.^10,23^ Beside efficiency of FR also depends upon the bio-anodic and cathodic potentials. Excess concentration of Fe^2+^ may result %OH loss and thereby gradual decline in degradation efficiency of the pollutant. Previously, the maximum degradation efficiency of azo dye was reported at 1.0 mM of Fe^2+^ in MEFS, however, further increase in Fe^2+^ concentration to 5 mM reduced degradation efficiency to 80.15% from 100%.^92^ Beside excessive Fe^2+^ addition may cause further operational costs^160^ due to great volume of sludge generation and its successive treatment. Some solid iron oxides such as α-FeOOH, FeO, Fe_3_O_4_, Fe_2_O_3_ have been considered as iron catalysts in Electro-Fenton process. The removal efficiency of POPs was related to release Fe^2+^ from its oxides into the solution for Fenton's reaction (Fig. 2C). Moreover, synthetic iron-containing nanoparticles (*e.g.*, nano-Fe_3_O_4_, α-Fe_2_O_3_ and Pd/Fe3O4) have also been considered for the treatment of POPs based upon electro-Fenton process.^59^

**Fig. 3:**
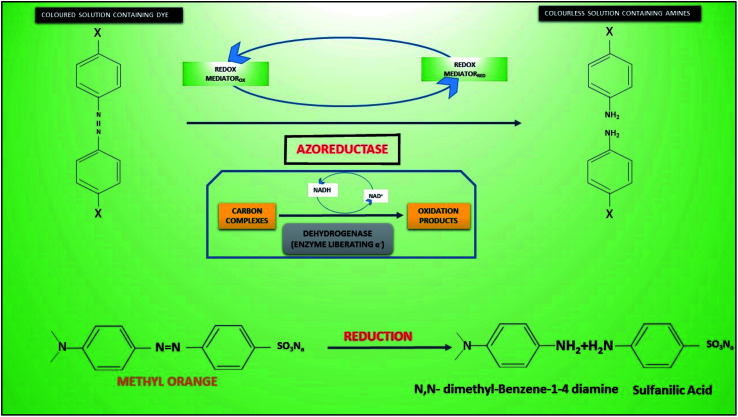
Proposed scheme of partial anaerobic degradation (decolorization) of methyl orange 7 (refabricated from ref. 169).

**Fig. 4:**
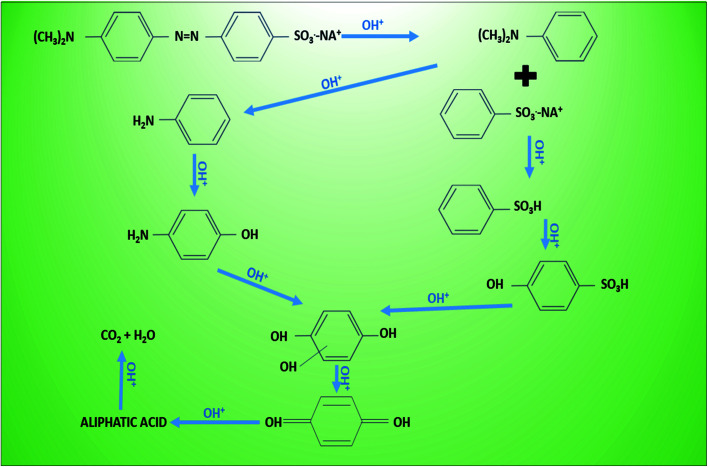
Degradation of methyl orange using hybrid advanced oxidation (refabricated from ref. 170).

Degradation of methyl orange using hybrid advanced oxidation (Fig. 4) suggested involvement of hydroxyl radical with amputation at electron-rich site of N=N (azo bonding),^163^ creating intermediates like dimethylaniline (a) and sodium benzenesulfonate (b) which then converted to aniline (c) and benzenesulfonic acid (d) respectively. Further hydroxylation of resulted conversation of aniline into hydroxyaniline (e) and benzenesulfonic acid into 4-hydroxybenzenesulfonic acid (f), and both these intermediates decomposed into hydroquinone (g) and then *p*-benzoquinone (h). The final aromatic end product is transformed into an aliphatic acid (oxalic acid or carboxylic acid) for complete mineralization into CO_2_ and H_2_O.^164^ Another sequential (symmetrical and asymmetrical) degradation of dye MO using hydroxyl radical demonstrated aromatic products such as hydroquinone and *p*-benzoquinone. Whereas aliphatic byproducts like succinic acid, malic acid, acetic acid and isobutaric acid are easily assimilate into TCA and other respiratory pathways. To attain high degradation performance in MEFS, high H_2_O_2_ concentration and production rate are obligatory. In MXCs-integrated systems containing both biotic and abiotic phases, the relationship between current, ARB, and the electrode revealed^123,165,166^ voltage (Ohmic) losses in the electrodes due to resistance at the interface of biofilm, anolytes/catholytes and electrodes, CEM/PEM membrane, and the cathode over potential (for H_2_ production or O_2_ reduction).^119^ The process conversion efficiency can be lowered by 100% due to losses at the bio-anode. In cathode chamber, the reaction mechanism is comparable with classical electro-Fenton process. However, in both cases H_2_O_2_ reacts with Fe^2+^ to yield hydroxyl radical that could be used for oxidation of dyes.^162^ The cathode potential is one of the key players in H_2_O_2_ production and it varies with different types and composition of cathodes. For example, a maximum H_2_O_2_ concentration can be achieved at cathode potential of −0.4 V using graphite cathode.^167^ Though, the maximum H_2_O_2_ concentration of 711.2 mg L^−1^ was witnessed under cathode potential of −0.85 V with 0.5 V in MEC using 3D electrodes as cathode.^168^ Apart from cathodic potential, cathodic material should be resistant to corrosion and should have good electrical conductivity.

## 4 Materials used in MEFS

### 4.1 Electrode material

**Fig. 5:**
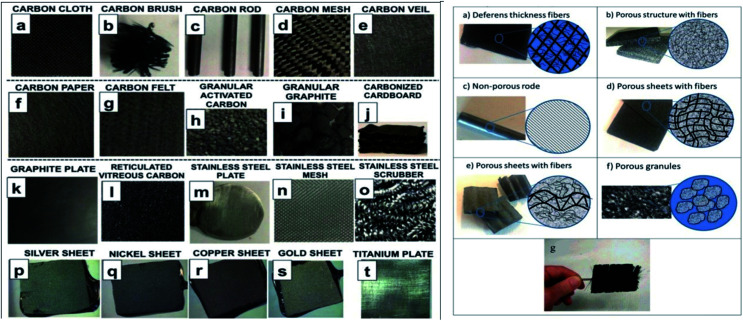
Different anode material used in MEFS (adopted from ref. 184 and 185).

Electrodes play an integral role on the efficiency of any electro-chemical or bio-electro-chemical cells. Electrodes types are characterized on the basis of their material, conductivity (electronic potential), reactivity, surface area, and their integration in the MEFS. Some of the electrodes have also been fabricated in laboratory.^171^ Typically in a MEFS, these electrodes are vital in terms of their compatibility with cellular organelles and exo-secretions (extra-polymeric substances = EPS) in order enrich biofilms of exo-electrogens on them.^172^ In many reactors, both electrodes *i.e.* cathode and anode were similar^173^ but in others cases they may be different.^174^ Carbon-based materials are preference in different MES including carbon paper, glassy carbon, carbon felt, carbon cloth, graphite plate, granule graphite, granule active carbon, graphite brush carbon mesh, carbon aerogels and reticulated vitrified carbon (Fig. 5). Most of the carbon materials, such as carbon paper, glassy carbon, carbon cloth, are composed of 2D structures that are plane with identical surface property and morphology. The electrochemical property of these pristine carbon materials can be improved effectively by proper surface treatment (acid, heat, amino gas, *etc.*) and modification with nanomaterials, conductive polymer, and immobilized electron shuttle that results insignificant improvement in performance of MES. Beside, these electrodes were modified with various types of materials like nanomaterials, activated carbon, metal (Pt catalyst) and *etc.* in order to improve their performance.^175^

3D anodes are grabbing substantial consideration for the improvement of profoundly efficient systems.^176,177^ These anodes have demonstrated 80–90% hydrogen recovery and COD removal efficiency.^153^ Additionally, layers of carbonized corrugated cardboard as anode material increased the current density by manifold.^178^ Furthermore, pre-treatment of anode with ammonia, phosphate, trace metals proved to be increasing; porosity (biocatalytic sites increases), conductivity, and particles capable of supporting biofilm growth.^179–181^ Whereas modification of C anode with polypyrole/anthraquinone-2,6-disulfonate cause the increased power density, redox properties and associated microbial-electo-catalysis of azo dye.^174^ Modification of carbon fibers with carbon nanotubes and polypyrole composite resulted in excellent biocompatibility and conductivity. Efficiency of modified anode in producing electricity is 2.63 times higher than that of unmodified ones, generating 1876.62 mW m^−2^ power density, and enhanced mineralization rate of orange II dye.^182,183^

### 4.2 Cathode and Fe forms and types

Cathode material should be porous and resistant to corrosion in order to support exchange of protons and electrons and interaction with oxidants. It is also usually carbon based due to less cost, good conductivity and stability.^162,177,186^ These include carbon brush, activated carbon, carbon fiber and modified CNT, carbon paper and cloth *etc.* that have been employed in MEFS.^177,178,187,188^ Production of H_2_O_2_ is vital in MEFS, and it then converted into highly responsive oxygen species such as ˙OH radicals to mineralize pollutants in cathode.^153,162,177,186^ Modified cathodes with C substrates with catalysts' layers and diffused (channeled) air supply proved to be highly effective in catalytic reduction of oxygen and H_2_O_2_ production.^177,184,189^ The iron source is one of the vital element to impact the performance of the MEFS. Usually, dissolve Fe^2+^ (Fenton) and sometimes Fe^3+^ (Fenton-like) salts have also been used in catholytes as catalysts for Fenton reaction in MEFS. Some researchers also have used iron alginate beads, scrap iron or iron plates as Fenton catalysts. Carbon-based modified cathodes with iron or transition metal oxides have also been preferred.^184^ Wang *et al.* reported the use of composite cathodes in MEFS for dye wastewater treatment. The cathode was fabricated by generating CNTs on SS316 stainless steel mesh with addition of iron phthalocyanine as catalyst, which resulted in enhanced current density and power of the system by 937 and 2594 times respectively along with 84.6% of dye decolorization in 12 h.^171^

Combination of both divalent iron and trivalent iron (*e.g.*, magnetite and ilmenite) has found more effective for Fenton reactions. These catalysts of iron were also used in EF studies in pure forms, doped with other cations,^59^ or mostly as composite electrodes.^190^ The usage of self-designed electrodes like Fe@Fe_2_O_3_/CF,^191^ γ-FeOOH/CF,^192^ carbon nanotube (CNT)/γ-FeOOH/CF, PPy/AQDS/CF^162^ and FeVO_4_/CF^191^ are providing iron catalyst as heterogeneous or homogeneous reactions has expanded the scope. Study showed that degradation rates of rhodamine B were 38%, 63% and 79% (15.3–30.1 a cm^−2^) with non-catalyzed CF (NCF), NCF with Fe^2+^solution (Fe^2+^/NCF) and Fe@Fe_2_O_2_/NCF composite cathodes at closed circuit (1000 Ω), however, it improved to 49%, 64% and 95% (current density of 43.7–65.2 μA cm^−2^) at short circuit (0 Ω external resistor), respectively, in 12 h. Among homogenous form of iron(iii) chloride hexahydrate (FeCl_3_·6H_2_O) and Iron(ii) sulfate heptahydrate (FeSO_4_·7H_2_O) iron salts are mostly used and relatively cheaper than heterogeneous salts.^40^ But they may cause Fe sludge generation and require adjustment of low pH for Fenton reaction thereby making system more costly. Contrarily, Fenton reaction with heterogeneous iron salts^35,162^ such as goethite (α-FeOOH), magnetite (Fe_3_O_4_), maghemite (γ-Fe_2_O_3_), clino-pyrrhotite (Fe_1−*x*_S) or hematite (α-Fe_2_O_3_) can be highly efficient for FR and catalysis of pollutants in mesophilic soil and wastewater.^193^ Moreover, coating of C felt and graphite cathodes with γ-FeOOH composite,^157^ pyrrhotite,^31^ and Fe@Fe_2_O_3_ (ref. 194) proved to be slowly leaching out and discharging ferric or ferrous ions in cathodic solution. Carbon aerogel with iron and copper proved to be another effective modification of cathode Fenton reaction enhancing WWT containing dyes (MB).^195^ Such modification help control release of iron and decreases sludge generation and management cost.^175^

## 5 Types and operation models of microbial-electro-Fenton systems

In the last two decades, developments in MEFS have provided considerable scope for innovative efficient solutions for wastewater treatment. Such green multifaceted technology has shown great prospects^7,22,23,196–198^ though they are still under stages of research and development for large scale application. Studies have reported treatment of dyes and pigments through different MEFS that has been implemented in different configurations, with different membranes, mostly proton exchange membrane (PEM), cation exchange membrane (CEM), and through their integration with biological degradation of dyes. In most of the studies, most commonly treated dyes in MEF systems include; Congo red, orange G, orange II, rhodamine B, methyl orange, reactive black 5 Lissamine green B and crystal violet. Comparatively, orange II (Acid orange 7) is the most studied azo dye in the said systems.

MEF technology typically evolved as an extended version of MFCs and MECs, where apart from anodic (anaerobic) oxidation of organic compounds additional cathodic oxidative detoxification and decomposition of certain pollutants is carried out through highly reactive hydroxyl radicals generated in Fenton reaction. However, previous WWT processes including batch, sequential or continuous flow reactors also helped in innovating MEF technology. Extensive studies of MEFS are underway toward development of new designs for achieving ideal system performance. MFC-Fenton system is though less efficient but cost effective in terms of electrical and H_2_O_2_ outputs associated Fenton reaction for dyes treatment.^30,154,199^ Development of single chamber rather than double chamber considerably improved the energy out of the MFC system.^200^ MFC powered EFS has also gained advancement through integration of super-capacitor for bio-electricity storage for subsequent use in Fenton reaction.^21,201^ Considering treatment efficiency on priority compared to electrical energy outputs, MEC based Fenton process has shown greater prospects. Such system is highly efficient for treating high load of contaminates and is equally good to treat domestic and industrial wastewater at the same time. Still this system need an additional input of minor energy (0.2–0.8 V) although, it is about 100 times lower than conventional EFS.^32^ But the possibility that lies in usage of single chamber MFC to empower a two-chamber MEC-electro-Fenton system for treatment of persistent pollutants is another good option to avoid any external input electrical energy for self-sustainable.^27^ Before seeking industrial scale applications there are several other challenges that need to be addressed.

Microbial Reverse Electro-dialysis Cells (MRECs), Automated Microbial Fenton Cell (AMFC) are the most innovative in MEFS.^32,183,202^ The development of continuous or semi-continuous mode of reactors is highly required in order to further enhance these technologies at large commercial scales relying on previously developed anaerobic and MFC/MEC based reactors. MEFS technology have offered considerable opportunity of treating two different substrates (pollutants) or wastewater in two separate chambers of the same reactor.^175^ They have been studied for the removal of natural (biopolymers) and synthetic/recalcitrant compounds including azo dyes (Congo red,^35^ methylene blue,^27^ acid orange,^173^ orange II, rhodamine B), pesticides, heavy metals, phenol, trimethoprim, caffeine, sulfamethazine, and ranitidine.^203,204^ The efficiency of systems above varies with electrode material, microorganisms, pH, current density, cathode potential, and concentration of ferrous/ferric ions their homogenous and heterogeneous forms.

### 5.1 Classification of microbial-electro-Fenton reactors

MEFS can be classified based upon their configurations and mode of operations. There are operated as (i) single cell [*e.g.* MFC or MEC or MREC], (ii) double cell [MFC-AFT],^29^ and (iii) hybrid or sequential [EF and degradation of microbes].^203^ Most of them run under batch mode and only few under continuous modes.^205,206^ Besides, there are mostly laboratory scale reactors, and few pilot scale with none large scale.^44^ Considering MFC batch reactors as the bench mark, several modifications have been done in its configuration and fabricating material converting it into more efficient wastewater treatment and energy generating technology.^207^ For instance, Modification of C anode with polypyrole/anthraquinone-2,6-disulfonate cause the power density to increase, redox properties and associated bio-electo-catalysis of azo dye.^174^ Modification of carbon fibers with carbon nanotubes and polypyrole composite resulted in excellent biocompatibility and conductivity. Efficiency of modified anode in producing electricity is 2.63 times higher than that of unmodified ones, generating 1876.62 mW m^−2^ power density, and enhanced mineralization rate of orange II dye.^183^

#### 5.1.1 Batch mode of operation

In batch mode, specified amount of waste liquors as anolytes and catholytes are treated for a stipulated period of time and then drawn out to revive the reactor.^208^ MFC or MEC reactors have been mostly used under batch mode reactors in various studies. Agitation is provided in the system using magnetic stirrer for the anolytes and catholytes.^23^ Aeration is maintained in a cathodic chamber by using commercial air pumps.^209^ Time span of treatment usually varies 2–10 days; however, it may exceed to 30 days. It purely depends upon the efficiency of the system with reference to specific waste type. For simple substrates generally it usually takes less time whereas for complex substrate longer time is required.^207^ However, major limitation of batch reactor is organic compound's mass transfer rate mass to microbes on anodic surface for biocatalysis and harnessing of electrons and protons to harvest cathodic H_2_O_2_ (ref. 50) for FR. However, depletion of nutrients in medium under batch mode may cause potential loses and inefficiencies in the systems.^210^Table 2 shows details on different batch or sequential mode MEF reactors with their operational conditions and efficiencies for dyes wastewater treatments.

**Table 2:** Removal of dyes in different (batch or sequential) MEFS

Reactor configuration	Anodic inoculum	Cathode material	Pollutants	Concentration	pH	Amendments	Power density	Removal efficiency	Operation time	Reference
MFC	*Shewanella decolorationis* S12	CNT/PTEF/γ-FeOOH	Orange II	35 mg L^−1^	7	Synthesis of H_2_O_2_ in MFC using cathode as spectrographically pure graphite (SPG)	25.13 mW m^−2^	100%	43 h	126
MFC	Anaerobic sludge	Graphite	Acid blue 113	300 mg L^−1^	3	MEF was operated with effluents of constructed wetland	3.5 A m^−2^	91.57%	43 h	94
Dual chamber MFC	*Shewanella decolorationis* S12	Ppy/AQDS/carbon	Orange II	10 mg L^−1^	7	Modified electrodes used to enhance performance	823 mW cm^−2^	97%	7 h	211
Dual chamber MFC	*Shewanella decolorationis* S12	CNT/γ-FeOOH	Orange II	10 mg L^−1^	7	Modified electrodes used in dual chamber MFC	230 W m^−2^	100%	14 h	92
MFC	Glucose	Spectrographic pure graphite (SPG)	OrangeII, Amaranth	75 mg L^−1^	7	Iron conc. Changes 1.14–3.43 mg L^−1^ & 0.1–1 mg L^−1^	25.13 mW m^−2^	82.59%	1 h	92 and 126
MFC	Anaerobic sludge	Graphite	Amaranth	75 mg L^−1^	3	Optimal cathode conditions were applied n cell for H_2_O_2_ production	42.6 Am	84.24%	2 h	167
MFC	Brewery wastewater	Fe@Fe2O3/NFC	Rhodamine B	15 mg L^−1^	3	The double chamber MFC was proposed by utilizing Fe@Fe_2_O_3_/carbon felt composite cathode	307 mW m^−2^	95%	12 h	195
MFC (H-type)	*T.versicolor S.oneidensis*	Graphite rod	Lissamine green B	10 mg L^−1^	2	Combination of fungus and bacterium used	1.2 W m^−3^	94%	9 h	212
MFC (H-type)	*T.versicolor S.oneidensis*	Graphite rod	Crystal violet	10 mg L^−1^	2	Combination of fungus and bacterium used	1.2 W m^−3^	83%	9 h	212
Hybrid reactor of benthic MFC and EF	Sewage sludge, marine sediments	Graphite sheet	Lissamine green B, reactive black 5, indigo carmine	10 mg L^−1^, 20 mg L^−1^, 50 mg L^−1^	7	Marine sediment microbial fuel cell used to drive external electrochemical and electro-Fenton processes	1033–1046 mV	100%	1 h	36
MFC (hybrid cell)	Sewage sludge	Graphite sheet	Reactive black 5	50 mg L^−1^	7.5	SMFC anode and electro-Fenton cathode connected with a salt bridge	1033 mV	88.2%	15 min	36
MFC (hybrid cell)	Sewage sludge	Graphite sheet	Lissamine green B	10 mg L^−1^	7.5	SMFC anode and electro-Fenton cathode connected with a salt bridge	1034 mV	98.2%	15 min	36
MFC (hybrid cell)	Sewage sludge	Graphite sheet	Crystal violet	5 mg L^−1^	7.5	SMFC anode and electro-Fenton cathode connected with a salt bridge	1046 mV	96.2%	15 min	36
MFC (hybrid cell)	Sewage sludge	Graphite sheet	Indigo carmine	20 mg L^−1^	7.5	SMFC anode and electro-Fenton cathode connected with a salt bridge	1045 mV	97.2%	15 min	36
MFC (hybrid cell)	Sewage sludge	Graphite sheet	Poly R-478	80 mg L^−1^	7.5	SMFC anode and electro-Fenton cathode connected with a salt bridge	1035 mV	19.1%	15 min	36
MFC	*Shewanella decolorationis* S12	Ppy/AQDS/carbon	Orange II	70 mg L^−1^	7	Modified electrodes used in dual chamber MFC	823 mW cm^−2^	100%	50 h	174
MFC powered advanced FS	Anaerobic sludge	Carbon felt	Acid orange 7	50 mg L^−1^	3	MFC was combined with Fenton-like technology to simultaneously generate electricity and degrade dye	15.9 W m^−3^	89%	60 h	204
MFC	Anaerobic sludge	Fe_2_O_3_/ACF	Methyl orange	5 mg L^−1^	2	Composite Fe_2_O_3_/ACF electrode was used	268.10 mW m^−3^	86.7%	12 h	213
MFC	Anaerobic sludge	Graphite rod	Congo red	100 mg L^−1^	7	MFC and a COR reactor were integrated together	808.3 mW m^−3^	90%	72 h	35
MFC	Domestic wastewater	FePc/CNT/SS316	Reactive black 5	50 mg L^−1^	7	Composite cathode is used	726.55 mW m^−2^	80%	12 h	171
MFC	Anaerobic sludge	Graphite rod	Acid blue113	100 mg L^−1^	3	Graphite cathode was treated with nitric acid	36.438 mW m^−2^	71.36%	12 h	17
MREC	Domestic wastewater	Graphite	Orange G	400 mg L^−1^	2	Salinity gradient energy drove the microbial-electro-Fenton process	2 A m^−2^	100%	10 h	32
3D-EF-MFC	Anaerobic sludge	Activated carbon	Methyl orange	100 mg L^−1^	3	MFC coupled with 3D electro Fenton technique	566 mW m^−3^	84%	72 h	183
MFC-MEC	Domestic wastewater	Graphite	Methylene blue	50 mg L^−1^	3	MFC as renewable power source used to power MEC-electro-Fenton process	50.1 mW m^−2^	97%	16 h	27
MFC power	Anaerobic sludge	Graphite plate	Acid orange 7	16 mg L^−1^	6	A novel heterogeneous EAFL system driven by MFC used	54.02 mW m^−2^	96.4%	2 h	214
MEF-COR	Glucose	Graphite rod	Congo red	20 mg L^−1^	7	Integration of MEF with catalytic oxidation system	808.3 mW m^−3^	90%	72 h	35
MFC-AFT	Anaerobic sludge	Iron plate with carbon paper	Acid orange 7	400 mL (0.16 M NaCl)	3	External addition of 2 mM H_2_O_2_ and 0.3 mW power output	0.27 mW	85%	—	173
Automatic MEFS	Electroactive biofilm	Carbon impregnated iron oxide	Acid orange 7	50 mg L^−1^	7	Microchannel-structured carbon enlivened with iron oxides utilized as electro-Fenton cathode	—	93–96%	24 h	202

##### 5.1.1.1 One step (*in situ*) integrated process

One step integrated process involves a single MFC/MEC or MREC reactor, generating electrical potential for cathodic Fenton process. It is classified into following types based upon their configurations or designs.

###### 5.1.1.1.1 MFC based electro Fenton system (MFC-EFS)

MFC-EFS is basically a two chamber MEFS with *in situ* FR system in cathode. It can mineralize biodegradable organics in anodic chamber, and recalcitrant organic pollutants (dyes, pesticides) in cathodic chamber through Fenton reaction (FR) or electro-chemical Fenton (EF) treatment shown in Fig. 7A.^174^ MFC-EFS reactors are cost-effective than Electro Fenton (EF) processes because they are energy generating rather than consuming. Besides they also generate quantifiable amount of hydroxyl radical from FR for decomposing pollutants.^17^

###### 5.1.1.1.2 Automatic microbial electro-Fenton system (AMEFS)

**Fig. 6:**
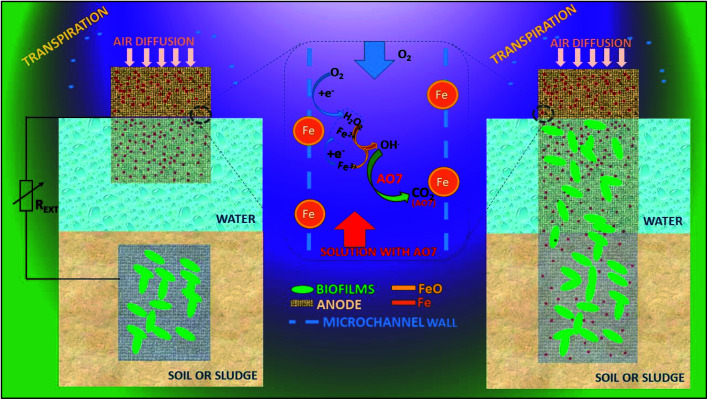
Automatic microbial electro-Fenton system (AMEFS) (refabricated from ref. 202).

A three-dimensional micro and macro-porous geometrical electrode provides large ratio of surface area to volume for biofilm development and electrolyte or substrate diffusion for efficient MEFS shown in Fig. 6.^215,216^ AMEFS is highly innovative and workable development in MEFS for *on situ* remediation of dyes and other related pollutants in water, soil or sludge. It is fabricated with microchannel-structured carbon impregnated iron oxides cathode for EF reaction and with an additional but similar anode for biofilm bio-catalysis in a two-electrode configuration connected by an external circuit. The second configuration of AMEFS includes a similar but single-electrode configuration serving both as bioanode and cathode. AMEFS is a spontaneously driven system similar to natural transpiration system in plants. The highest degradation efficiency (93–96%) at 50 ppm is generated at the condition of short-circuit with two-electrodes and that was comparable to single-electrode configuration. Such a system can be easily installed for sludge and soil purification by directly inserting microchannel structured carbon electrodes in the contaminated site or reactor.^202^ Furthermore AMEF is equally good to operate under batch or continuous mode operations.

###### 5.1.1.1.3 Other novel designs

**Fig. 7:**
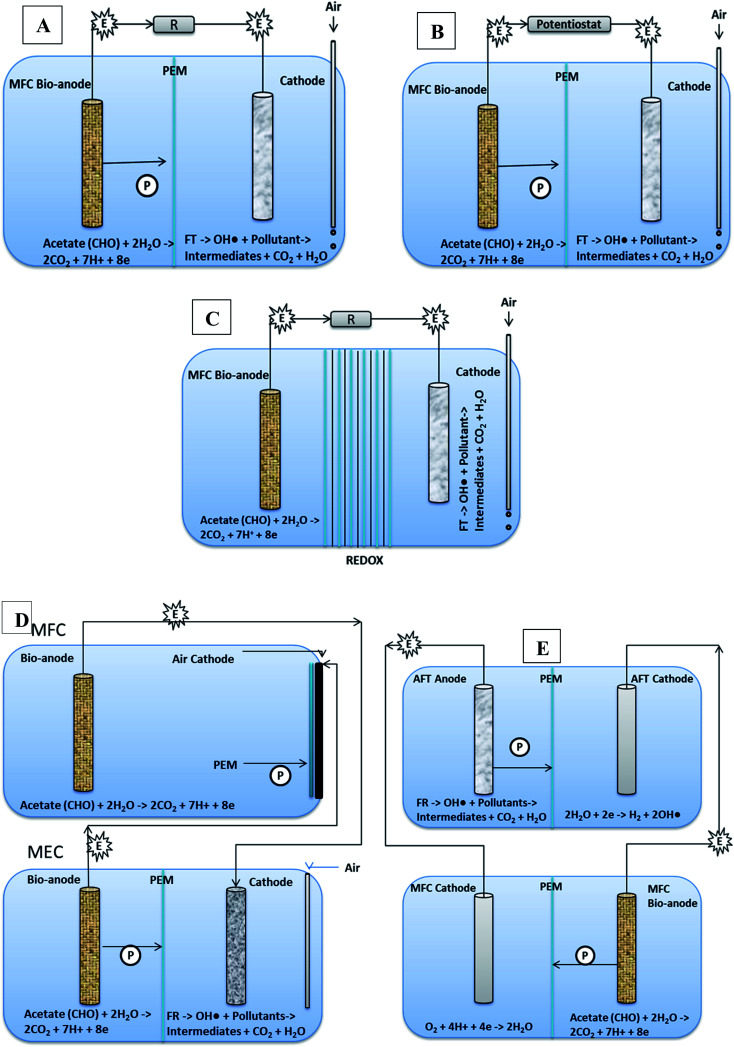
Removal of dyes in different batch MEFS (A) MFC-electro Fenton system (B) MEC-electro Fenton system (C) MREC-electro Fenton system (A–C single cells) (D) MFC-MEC-electro Fenton system (double cell) (E) MFC assisted AFT (double cell).

MECs an extended version of MFC with small input of charge (0.2–0.8 V) (Fig. 7B) has proved to be yielding H_2_O_2_ several time higher than MFCs. Therefore, MEC electro Fenton reactor can be suitable for treating wastewater that contains comparatively excessive loads of pollutants in both anodic and cathodic chambers with remarkably lower energy consumption than classical electro Fenton process.^217^ This system was efficiently used for azo dye WWT.^32^ Besides, it uses (approx low cost 25.93 kW h kg^−1^-TOC) an average of 75% (17 fold) less energy as compared to the regular electro-Fenton process (45.8–865 kW h kg^−1^ TOC).^218,219^ The degradation efficiency of TOC was found to be 93.1%. The larger removal at the short circuit leads to larger current density of 43.7–65.2 μA cm^−2^.^195^

MFC-3D EF is originally an MFC reactor but filled with granular AC either in both anode and cathode chambers or in cathode chamber as electrodes. The bed electrodes played an arbiter job in cathode and anode chambers, elevating the output voltage and power density, and lost cause internal impedance to get lowered. Dye evacuation efficiency is observed to be 84%.^183^

Microbial reverse electro-dialysis cells (MREC) is a novel hybrid bio-electro-chemical system for efficient H_2_O_2_ production through *in situ* electric energy generation by the microbial oxidation of biodegradable matters and salinity-slope that is between different fresh and salt channels and does not require power input (Fig. 7C). The cathodic and anodic chambers are separated with RED stacks and the reaction of cathode is not affected by the continuous flow of wastewater in anode chamber.^32^ It proved to be highly efficient in comparison with MFC and MEC with dye removal efficiency up to 99%. Moreover, the cost of the treatment proved to be lower (25.93 kW h kg^−1^ TOC) by ≥ 45% than other Fenton processes (45.8–865 kW h kg ^−1^ TOC).^218,219^

Using MFC as renewable energy source to power MEC Fenton process (Fig. 7D), electricity can be saved. But removal efficiency of pollutants and power density is found to be low as compared to traditional Fenton processes.^27^

MFC-AFT is a two cells system; where anodic Fenton reaction based treated of pollutants occurs in chemical cell supported by electronic energy harnessed from bio-anodic compartment of another MFC (Fig. 7E). Kinetic studies indicated that this system had higher pseudo-first order rate constant than other conventional Fenton processes and 87% of the dye was degraded in AFT system within 15 minutes. According to electrochemical analysis, the corrosion of iron was not hindered by dye. It was also revealed that MFC power density was improved by increasing dissolved oxygen in cathode compartment, which resulted in increased degradation of dye.^173^

##### 5.1.1.2 Sequential or hybrid process

**Fig. 8:**
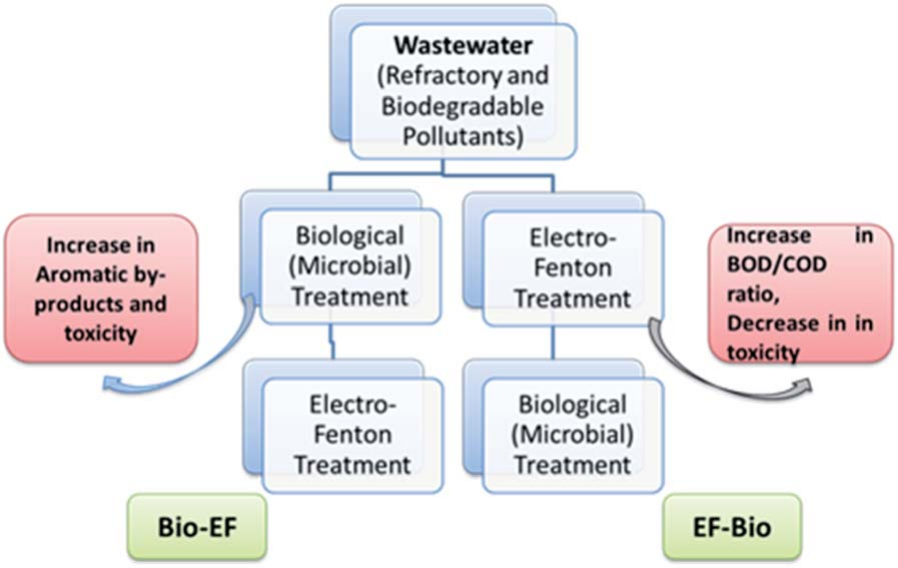
Sequential treatment of refractory compounds (dyes) through biological and EF/EAOP.

Sequential MEFS is a two-step sequential treatment of refractory compounds through pre or post EF oxidation with biological method in order to achieve a comprehensive efficiency shown in Fig. 8. The span of EF is minimized in order to keep the system cost effective. However, the type of effluent and its priority pollutant helps in deciding whether EF should be used as pre-treatment or post-treatment in conjugation with biological methods. EF as pre-treatment, involves hydroxyl radicals for oxidative mineralization of refractory compounds or into aliphatic by-products that may be sometimes toxic.^220^ Later biological process completely removes any remaining short chain compounds.^159,221,222^ EF-microbial oxidation proved to be effective treatment strategy for real textile wastewater. If EF is applied as post treatment then it helps complete decomposition of toxic and mutagenic aromatic by-products of compounds like azo dyes such as alpha-napthol or sulfanilic acid that are stable specifically under anaerobic biological condition.^21,223^ Selection of treatment strategy still greatly depends upon the nature of dyes. Previously, an EF-biological (aerobic) (yeast) process helped to degrade reactive black 5 dye 91%.^224^ EF and aerobic-microaerophilic process when combined removed almost 85% color and COD with 56% TOC of textile wastewater. Whereas, 52.7% color, 82.5% COD, and 41% TOC were observed when EF-aerobic microbial process was used. Moreover, dyes removal rates improved to >95% with 85% of TOC in a bubble column MFC reactor coupled *ex situ* EF process.^36,153^ The dyes wastewater usually contain many organic compounds beside of dyes. Some pretreatment *e.g.* coagulation, precipitation may be essential to improve dyes removal. Therefore, integrated process based on MEFT would be useful for dye wastewater treatment. Afanga *et al.* reported the sequential treatment of dye wastewater employing the integrated coagulation-electro Fenton process. The system achieved remarkable removal of organic matter along with dye degradation. The results revealed the removal of COD, TOC, and TSS up to 97%, 98% and 98% respectively.^225^

#### 5.1.2 Continuous mode of operation

In continuous mode reactors, the substrate (anolytes or catholytes) is continuously charged along with discharge of products simultaneously during upstream and downstream processing. Such systems are more dynamic and overcome the issues of mass transfer rates (coefficient) and nutrients depletion^226,227^ through constant flow and agitation provided.^23^ Current density obtained by continuous MFC was 2 times more than that obtained from MFC operated under batch mode.^207^ Continuous mode has higher efficiency in mass transfer and heat transfer and are less spacious than batch mode.^228^ In addition, continuous operation allows the usage of extreme conditions that aids to improve yield, continuous can allow up to 300° and 30 bar of temperature and pressure. Moreover, continuous process is easier, flexible, and faster to scale up than that of batch process.

**Fig. 9:**
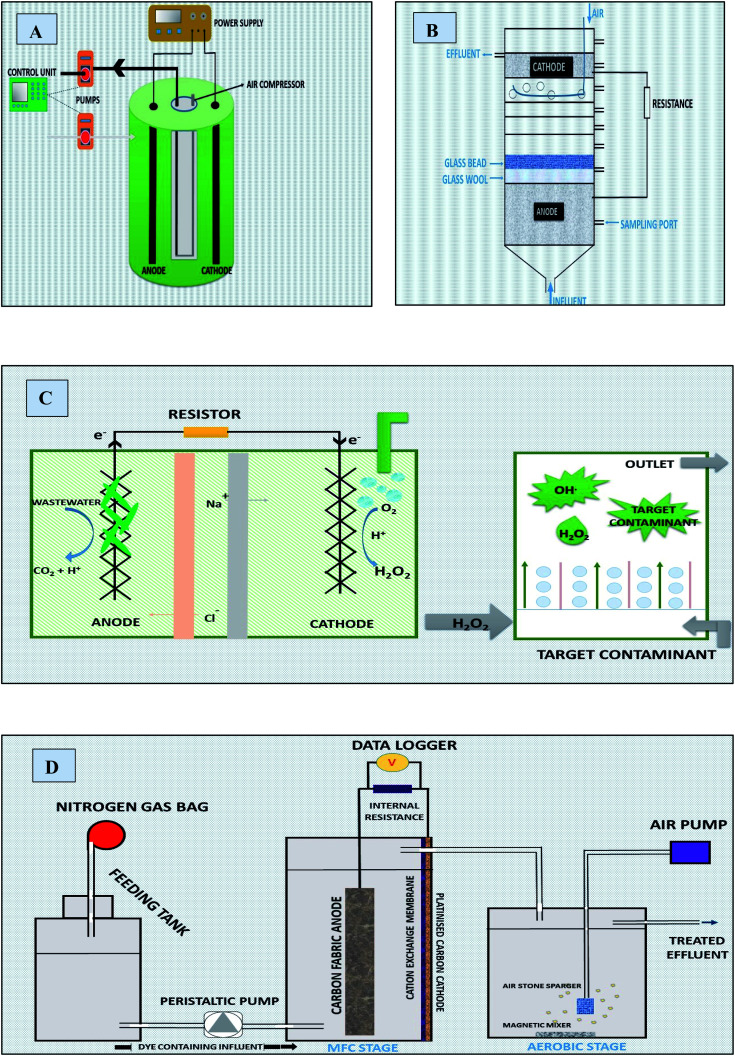
Continuous operated reactors (A) schematic diagram of Airlift continuous reactor with electro Fenton setup (refabricated from ref. 229) (B) schematics diagram of mediator-and membrane-less MFC column reactor (refabricated from ref. 240) (C) microbial desalination reactor (refabricated from ref. 175) (D) schematic of integrated MFC-aerobic system (refabricated from ref. 153).

Previously, different continuous flow reactors *viz.* Airlift^229^ Fluidized bed reactor, bubble reactor, Tubular,^230^ Plug flow,^226,231^ MFC, MDC and MEC [MFC-MEC, MFC-membrane^232^ were used based upon either microbial or electro-chemical processes for treatment of different pollutants (Table 3). Such reactors provided a solid platform for development of any continuous MEF reactors. Fig. 9 shows the various continuous flow reactors.

##### 5.1.2.1 MFC and MEC

Earlier MES studies reported both partial and almost complete azo dyes degradation (*e.g.* acid orange-7, *etc.*)^233^ in batch and continuous flow integrated MFC (-aerobic system) and MEC (-membrane).^153^ Single-chamber MFCs or membrane-less MFCs are favored over double chamber even for large scale WWT.^234^ Moreover, single chambered MFC tubular or inner circular electrodes proved to be superior for efficient treatment of azo dyes. Integration of Fenton process in MFC holds the greater potential of efficient degradation and detoxification of the dyes through maintaining maximum cathode half-cell potential for higher production of H_2_O_2_. Recently, a pilot scale (20 L) two chamber rectangular MEF reactor was developed to treat dyes WWT under continuous mode.^44^

##### 5.1.2.2 Microbial desalination cell (MDC)

A microbial desalination cell (MDC) is one of the most innovative modified version of MFC containing a desalination compartment sandwiched between CEM and AEM for separating it from cathode and anode chambers respectively. MDC is capable of simultaneous WWT and desalination with minor input of energy.^173^ Charge balance is maintained by salt ions migration from desalination chamber to cathode and anode by ion exchange membranes.^117^ MDC-MEF hybrid system, comparatively a novel approach, could be employed for the efficient removal of dyes from wastewater. Huang *et al.* reported application of MEF integrated MDC for increased degradation of methylene blue. The system reached maximum; power density of 566 mW m^−3^ and H_2_O_2_ of 24.07 g per m^3^ per day with degradation efficiency up to the 82.8% under the influence of Fenton reaction. Without which the system was capable to attain only 53% efficiency.^183^ Comparison in performance of a MEFS was investigated by switching MDC batch mode to continuous. System demonstrated a maximum power density of 15.9 W m^−3^ in continuous and 13.9 W m^−3^ in batch mode. Additionally, 83.7% of COD was eliminated in the continuously fed system at two days hydraulic retention time, and it was 13.8% more than that obtained under a two days batch system. It was demonstrated that enrichment of ARB and high mass transfer under continuous flow and feeding condition might have improved the system performance.^207,228^

**Table 3:** Characteristics of continuous mode reactors

Reactor configurations	Inoculum	Pollutants	Operational parameters	Hydraulic Retention time	Applied-voltage/current	Efficiency	References
Two chambers having 10 L each, anode: Carbon fiber brush, cathode: graphite plate	Domestic waste water	Methylene blue	Aeration rate of 350 mL min^−1^, 20 mg L^−1^, pH 2, Fe^2+^ of 0.2 mM, applied voltage 0.4 V	28 h	0.2 V	95% dye decolorization 89% TOC removal	44
SMFC, electrodes: Graphite sheet	Marine sediment	Lissamine green	Na_2_SO_4_ 0.01 mol L^−1^, iron concentration 150 mg L^−1^	1 h	—	97–99% decolorization	36
Cylindrical reactor containing two electrodes at the center, cathode: modified graphite with carbon nanotubes, anode: Graphite	Dye solution	C.I. acid red 14 (a), C.I. acid blue 92 (b)	NaCl: 1 g L^−1^, 0.05 mM Fe^3+^, pH: 3, aeration 10 mg L^−1^ dye, effective volume 1 L, flow rate: 0.33 L h^−1^	—	0.14 A	86.78% COD removal in 60 min, pollutant degrading efficiency 91.22% (a) 93.45% (b)	235
Microbial fuel cell MFC (H type)	*T. versicolor and S. oneidensis MR-1*	Lissamine green B, crystal violet	Graphite rod used as anode, dye conc. 10 mg L^−1^, pH 2, iron concentration 150 mgL^−1^	—	—	94% Lissamine green B, 83% crystal violet after 9 h treatment time	212
Fluidized bed reactor, cathode: nickel foam layered with iron-chitosan, anode: graphite sheet	Dye solution	Lissamine green B	pH 2, 0.15 L working volume, 0.01 M, Na_2_SO_4_, 100 mg L^−1^ dye, mixing by continuous air flow at 0.15 vvm^a^	45 min and 90 min	5 V	75% TOC removal, dye degradation efficiency 95%	236
Cylindrical glass reactor, electrodes: graphite bar	Dye solution	Lissamine green B (a) reactive black 5 (b)	pH = 2, 22 °C, catalyst 115 g Fe alginate gel beads made of sodium alginate, Bacl_2_, Fe_2_(SO_4_)_3_, air bubbling near cathode at 1.5 L min^−1^, working volume 1.5 L	6 h (a), 12.5 h (b)	3 V	TOC removal 81% (a), % (a), 87% (b)	229
Cylindrical glass reactor. Electrodes: graphite sheet	Dye solution	Lissamine green B (a) azure B (b)	Catalyst: 8.69 g Fe alginate beads, air bubbling near the cathode at 1Lmin^−1^, working volume 0.15 L, pH=2	30 min	14.19 V	TOC removal 93% (a) 89% (b)	237
Bubble reactor formed of cylindrical glass, electrode: graphite	Dye solution	Lissamine green B, methyl orange, reactive Black 5, Fuchsin acid	0.04 M Na_2_SO_4_, 8.5 mg L^−1^ (LGB), 1.5 mg L^−1^ (MO), 70 mg L^−1^ (RB5), 15 mg L^−1^ (FA), pH=2, bubbling compressed air at L min^−1^, 0.675 L working volume	21 hour	15 V	47% TOC removal, 43% dye removal	238
Bubble glass column reactor, electrodes: graphite	Textile wastewater	Rhodamine B	pH 3, catalyst: FeCl_3_ at 5 mg L^−1^, 3 L of electrolyte at 10 mL min^−1^	8 h	3.5 V	98% dye - removal	239

##### 5.1.2.3 Hybrid cell: sediment MFC (SMFC) or benthic MFC (BMFC)

Benthic or Sediment MFC (BMFC or SMFC) assisted FR and BMFC-FR hybrid processes also highlights importance of continuous or semi-continuous cost effective wastewater treatments.

## 6 Feasibility of scaling up of MEFS and cost benefit analysis

Current developments on MEFS propose innovative energy efficient solutions for dyes and industrial WWT in near future.^21^ Most of the MEF studies that are conducted so far are at lab scale, relying upon MFC and MEC configurations.^44^ The greatest driver in up-scaling of MEFS would be MFC/MEC based multifaceted reactors that work on the principles of harnessing renewable energy from WWT to minimise the capital cost.^59^ Initial advancement in MFC or MEC at pilot scale integrating EF reactors by addressing previously reported drawbacks could be the benchmark for further developments. Nevertheless, some reports outlined the practicality of the said technology for direct/or indirect power generation from wastewater at volumes exceeding those in lab scales MEC^241^ and MFC.^176,242–245^ Comparatively, WWT capacity of MEC based MEF system proved to be much greater than that of MFC based system.^27^ A very recent study mentioned up-scaling of MEC (0.4 V) based MEFS of 20 L for the treatment of dye WW using C fibre brushes as anode and graphite plate and silver/silver chloride as cathode with Fe^2+^ 0.2 mM and Na_2_SO_4_ 50 mM. Almost 100% removal of methylene blue dye was observed after 8 hours treatment. Likewise the dyes and TOC removal rates were 100%, 70–90% in case of Meldola's blue (MeB), Toluidine blue (TB), Orange G (OG) and Rhodamine 6G (Rh6G) and Rhodamine B (RhB) after 28 hours respectively^44^ Overall, complete mineralisation of dyes was significantly less compared to their decolorization and it has been related to their complex aromatic structure and high molecular weight. Other studies on lab scale MEFS reported that for MB removal, the *K*_app_ was 0.43 h^−1^ and *K*_TOC_ was 0.22 h^−1^, while compared to Orange II, *K*_app_ was 0.212 h^−1^ and *K*_TOC_ was 0.0827 h^−1^.^174^ Thus, the results explained that scaled-up MEFS has higher efficiency than that of MEFS operated at lab scale for the treatment of dyes containing wastewater.

Prior studies have revealed that MEF technology is a cost-effective substitute to traditional EF technology for the treatment of dye wastewater with energy consumption of only 25.93 kW h (kg TOC)^−1^.^32,39^ But for large scale application, cost benefit analysis of MEF systems is vital for its feasibility in dyes and industrial WWT.^44^ MFC based MEFS are facing technical and cost challenges for commercial viability at large scale. Moreover, improving the performance and reducing the capital costs of reactor and its operation need to be addressed. System scaling-up requires extensive studies on the reactors' designs and material and operational variables, typically related to continuous *in situ* regeneration of catalyst *i.e.* Fe^2+^, and H_2_O_2_ in order to prevent sludge formation.^21^ So, primarily, the design of bio-reactor is extremely important for optimum functioning and then up-scaling. Initial modeling through computer simulated program of MEFS is highly important as an effective tool for reactor configuration.^246^ The major cost based variables include electrodes and membranes (CEM/PEM). Specifically, the cost of the membranes sometimes cost an about 38% of the total cost of the reactor. For instant, Nafion membrane cost around 2500 $ per m^2^.^247^ And is 37 fold higher than sulfonated biochar (SBC)-600 with PVA membrane costing 77 $ per m^2^. Contrarily, per unit cost of SBC-600 membrane for proton conductivity was 0.42 S per cm per $ and that proved to be 32 times greater than Nafion membrane (1.31 × 10^−2^ S per cm per $). Likewise, the estimated standardized energy reclamation for SBC based MFC was much lower *i.e.*, 0.014 kW h m^−3^ (6.356 kW h kg COD^−1^) than Nafion based MFC (0.024 kW h m^−3^ (9.59 kW h kg COD^−1^) system.^248^ Additionally, GORE-TEX membrane has been reported quite cheaper and costing an about 21 $ per m^2^ and besides, the PVA modified membranes has been reported equally cost-effective.^247^ The electrodes do a crucial job in electricity generation and a main factor in deciding the implementation of the MFC technology at a large level. Different materials ranging from non-corrosive stainless steel to versatile carbons have been investigated as anodes in different structures and shapes.^249^ The cost of C clothes varies from $ 100–1000/m^−2^, Still, cheap carbon mesh based electrodes costing an about $10–40/m^−2^ can also be another good alternative to of carbon cloth.^250^ Moreover, the cost of cathode increases when it is modified metal catalysts [*e.g.*, FeSO_4_ cost = $13782/kg and FeOOH cost = $ 826/kg]^251^ to apply in MEFS. In this case possibility of using scrap iron waste and the wastewater originating from steel and mining industry could be a good source of iron in cathode for Fenton reaction.^251,252^ In the similar situation, usage of non-polished graphite over carbon might increase the microbial adhesion over anode and will be cost-effective for huge-scale operation. Recently cheaper such as activated carbon and metal mesh showed oxygen reduction performance.^247^ Usage of graphite coated with metal as cheaper electrode material and carbon felt results in better performance and electron transfer because of their low cost, good conductivity and huge surface area in contrast to other expensive materials.^253–255^ Bio-electricity production in system (*e.g.*, MEC-MEF) through microbial bio-catalysis of organic compounds cut down energy budget by almost 40–50%.^252^ When the MEF process run without external energy than the normal electrical energy budget was 11 526.6 kW h (kg TOC^−1^) with respect to TOC removal costing US$ 2420.59. The higher cost relates to higher energy consumption and lower efficiency in MFC based MEF process requiring more reaction time and aeration costs. However, MEC based system requires a small input (0.2–0.8 V) of electricity in order to generate H_2_O_2_ in cathode at much high rate as compared to the MFCs. The production of H_2_O_2_ requires much lower energy *i.e.*, 0.93 kW h kg-H_2_O^−1^ with a cost price of US$ 0.195 in MECs compared to traditional electrochemical process^20,27^ Still only small amount of electrical energy was directly applied *i.e.* 5.96 kW h (kg TOC)^−1^ with a cost of US$ 1.25,^44^ and it was considerably low in comparison to the traditional EF processes.^256,257^ Besides, the energy budget of MEC based MEF system is highly economical considering the *in situ* bioelectricity generation by microbes that reduces the electrical energy consumption [0.728 kW per h per kg for aniline) may be hundred times lower than conventional electro Fenton process (74 kW h kg_aniline_^−1^).^217^ The approximate capital cost of MES (MFC and MEC) reactors were reported to be $100,000/ton COD × day and $1220/m_a_^3^.^258,259^ MREC-Fenton system has been reported to treat azo dyes (Orange G) containing wastewater with a cost of US$ 5.44 for 25.93 kW h (kg TOC)^−1^ which is very less as compared to traditional EF processes costing US$ 9.6–179.76 for 45.8–865 kW h (kg TOC)^−1^.^21,218,219^ The substitution of electrical energy from power grid to the renewable salinity gradient for direct driving of Fenton process is the main advantage of MREC-Fenton system.

Previously MEFS were mostly tested with artificial WW, so there is a need to operationalize the reactors with real wastewaters. At laboratory stage it was 0.25 kW h m^−3^ or 0.40 kW per h per kg of COD/0.18 kW h m^−3^ and 0.12 kW per h per kg of COD in terms of energy consumption for the reactors with pure substrates such as acetate and glucose respectively and was significantly higher compared to domestic [0.04 kW h m^−3^ or 0.17 kW per h per kg of COD] or industrial [0.01 kW h m^−3^ or 0.04 kW per h per kg of COD] wastewaters.^243^ But systems performance was still lower in power density that could be insufficient enough for large scale industrial plants.^260^ So there is a need to optimize the MEFS with real WW to apply it at large scale. Moreover, life cycle assessment of the most efficient microbial-electron-Fenton systems is vital to operationalize them at commercial level.^261^

## 7 Conclusions

Microbial electro Fenton technology for dye wastewater treatment have emerged as scientific inquisitiveness and are proved to be innovative, multidimensional approach that can be operated under batch and continuous mode where chances of treating domestic and dye wastewater can go side by side. Comparatively, the system is preferable to other methods due to its higher efficiency in terms of time and treatment. Characteristics, classification, environmental fate, toxicity and degradation mechanism of dyes have been assessed critically. Various types and operational models of MEFS for dyes wastewater treatment have also been reviewed. MFC being a platform technology has been greatly explored. In past, MFC based MEFS studies have shown to reduce the operational time from days to hours, even minutes, along with mineralization rate up to 90% and even above in case of dyes. Being an innovative technology, dual advantages of electricity production along with dye decolorization simultaneously have also been reported by *in situ*-designed MFC based Fenton process and generated electricity has been utilized to run *ex situ* Fenton process in continuous and batch operations. The scope of technology has broadened as it is heading toward bio-monitoring, paving way for upcoming competitive approaches. Despite promising, it still faces several challenges of reactor configuration, high electrode/membrane cost, iron toxicity and information on large scale application. Therefore, extensive research and investigation is indispensable for future exploration and upgradation.

In future studies, there could be an opportunity of replacing chemical catalyst by biological catalysts produced by microorganisms in cathode chamber to accelerate and improve the pollutant treatment. This can mitigate the predicament of iron toxicity along with the provision of other beneficial opportunities.

## Conflicts of interest

There are no conflicts to declare.

## Supplementary Material
